# Ozone stress response of leaf BVOC emission and photosynthesis in mountain birch (*Betula pubescens* spp. *czerepanovii*) depends on leaf age

**DOI:** 10.1002/pei3.10134

**Published:** 2024-02-04

**Authors:** Erica Jaakkola, Heidi Hellén, Stefan Olin, Håkan Pleijel, Toni Tykkä, Thomas Holst

**Affiliations:** ^1^ Department of Physical Geography and Ecosystem Science Lund University Lund Sweden; ^2^ Atmospheric Composition Research Finnish Meteorological Institute Helsinki Finland; ^3^ Department of Biological and Environmental Sciences University of Gothenburg Gothenburg Sweden

**Keywords:** Betula, oxidative stress, ozone, photosynthesis, sub‐arctic, volatile organic compounds

## Abstract

Oxidative stress from ozone (O_3_) causes plants to alter their emission of biogenic volatile organic compounds (BVOC) and their photosynthetic rate. Stress reactions from O_3_ on birch trees can result in prohibited plant growth and lead to increased BVOC emission rates as well as changes in their compound blend to emit more monoterpenes (MT) and sesquiterpenes (SQT). BVOCs take part in atmospheric reactions such as enhancing the production of secondary organic aerosols (SOA). As the compound blend and emission rate change with O_3_ stress, this can influence the atmospheric conditions by affecting the production of SOA. Studying the stress responses of plants provides important information on how these reactions might change, which is vital to making better predictions of the future climate. In this study, measurements were taken to find out how the leaves of mature mountain birch trees (*Betula pubescens* ssp. *czerepanovii*) respond to different levels of elevated O_3_ exposure in situ depending on leaf age. We found that leaves from both early and late summers responded with induced SQT emission after exposure to 120 ppb O_3_. Early leaves were, however, more sensitive to increased O_3_ concentrations, with enhanced emission of green leaf volatiles (GLV) and tendencies of both induced leaf senescence as well as poor recovery in the photosynthetic rate between exposures. Late leaves had more stable photosynthetic rates throughout the experiment and responded less to exposure at different O_3_ levels.

## Graphical abstract

Impacts of elevated tropospheric ozone on mountain birch leaves reveal higher vulnerability in young leaves compared to old ones. The leaf recovery after high concentration exposure is affected by a lower photosynthetic rate and increased stress‐related sesquiterpene emissions.

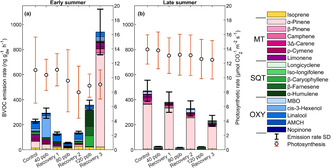

## 1 INTRODUCTION

Biogenic volatile organic compounds (BVOC) are organic hydrocarbons that are produced and emitted into the atmosphere from vegetation and other living organisms. Plants emit around 760 TgC of BVOCs into the atmosphere each year through physiological processes such as photosynthesis and respiration (Fitzky et al., 2023; Ghirardo et al., 2011; Guenther et al., 1993; Maffei, 2010; Sindelarova et al., 2014). Since BVOCs are produced through light‐ and temperature‐dependent processes, they are affected by changes in these factors as well as by additional abiotic and biotic factors causing plant stress. As a defense against stresses, plants respond by altering their constitutive BVOC emissions, either by changing their compound composition or even by inducing emissions of compounds they have not emitted before (Holopainen & Gershenzon, 2010; Loreto & Schnitzler, 2010; Sharkey et al., 2008). Once emitted, either constitutively or induced by plant stress, BVOCs are part of several chemical chain reactions in the atmosphere where terpenoids like isoprene, monoterpenes (MTs), and sesquiterpenes (SQT) are highly reactive. The emitted compounds become part of gas‐phase oxidative reactions with air pollutants and greenhouse gases like tropospheric ozone (O_3_) that are formed by photochemical reactions in the presence of nitrogen oxides (NO_x_). The gas‐phase reactions from the BVOCs are affecting the properties of the atmosphere by enhancing the production of secondary organic aerosols (SOA; Laothawornkitkul et al., 2009; Monson & Baldocchi, 2014), leading to changes in the energy budget of the earth by scattering the incoming radiation. Oxidation products from BVOC can also act as cloud condensation nuclei, enhancing cloud formation and affecting the energy budget similarly to SOA (Monson & Baldocchi, 2014; Paasonen et al., 2013; Scott et al., 2018). A shift in the emission of BVOCs can thus have large implications for atmospheric chemistry and radiative forcing.

Not only do induced BVOC emissions impact the atmosphere, but they also have high metabolic costs for the plants themselves (Keeling & Bohlmann, 2006). The high metabolic costs are evident from plant stress from elevated chronic tropospheric O_3_, resulting in degrading crop yields and decreased forest growth from losses in photosynthetic carbon assimilation (Loreto & Schnitzler, 2010; Mills et al., 2011). This aligns with responses seen in BVOC emissions to abiotic stress from O_3_ exposure, where, depending on the plant species and its O_3_ tolerance, emissions were both induced or reduced (Esposito et al., 2016; Kask et al., 2021; Peron et al., 2021; Vitale et al., 2008). Exposure to O_3_ for extended periods also led to changes in the BVOC blend, mainly with induced MT emissions (Loreto et al., 2004; Moura et al., 2022; Rinnan et al., 2005). Induced emissions of SQT were also found to be positively correlated with increases in O_3_ concentration (Bourtsoukidis et al., 2012; Moura et al., 2022). Apart from chronic O_3_ exposure, it is also important to consider short‐term ozone exposure at high doses (acute stress) that can cause major harm to plants by inducing cell death and altering metabolic profiles without visual damage (Vainonen & Kangasjärvi, 2015). Some tree species are more tolerant to O_3_, often related to leaf glandular trichomes that have been proven to protect the plants against O_3_ stress by exuding metabolites that deplete O_3_, where the level of protection is correlated with the density of the trichomes (Li et al., 2018; Mofikoya et al., 2018; Valkama et al., 2004). Leaf age has also been proven to be important for O_3_ protection. Younger leaves tend to have better antioxidant protection against O_3_ damage compared to older leaves in terms of foliar injury and increases in metabolite concentration (Wedow et al., 2021; Zhang et al., 2010).

Leaf age also matters in terms of BVOC emissions; higher constitutive emissions were found from young leaves of downy birch (*Betula pubescens*) compared to older leaves (Hellén et al., 2021). In general, the BVOC emission profile from birch trees is dominated by MT and SQT with little contribution from isoprene (Haapanala et al., 2009; Hakola et al., 2001; Hellén et al., 2021). The BVOC emission profile of birch was, however, altered by O_3_ exposure; potted birch saplings increased their overall emission rates when exposed to concentrations >80 ppb for long periods (Carriero et al., 2016), while MT emissions were decreasing with 150 ppb short‐term exposure (Timkovsky et al., 2014). Birch trees are considered an O_3_‐sensitive species where high exposure has affected tree growth negatively and promoted leaf senescence (Karlsson et al., 2003; Maurer & Matyssek, 1997; Saleem et al., 2001). The sub‐Arctic mountain birch (*Betula pubescens* ssp. *czerepanovii*) can, in particular, be further sensitive as the protective glandular trichomes on mountain birch leaves have a lower density compared to other common birch subspecies (Valkama et al., 2004). Mountain birch emits a wide range of BVOCs constitutively; between 12 and 4000 ng g_dw_^−1^ h^−1^ MT and 5 to 2700 ng g_dw_^−1^ h^−1^ SQT were reported as emission potentials at 20°C (Ahlberg, 2011; Haapanala et al., 2009).

Given the impact that BVOCs have on the chemistry and physics of the atmosphere and the resulting effects on climate change, it is crucial to understand how elevated O_3_ exposure affects leaves, especially in areas where the leaves have not adapted to high O_3_ levels yet. Mountain birch grows in the sub‐Arctic part of Sweden, an area not experiencing as high O_3_ exposures as the rest of northern Europe (Klingberg et al., 2019). The Arctic air quality is, however, expected to be degraded by increased ship traffic, causing increases in O_3_ concentrations of approximately 5% by 2030 (Gong et al., 2018). This makes it a relevant area for studying how elevated concentrations of acute O_3_ exposure are affecting birch leaves that have not yet adapted to such conditions. There is also a knowledge gap in how mature birch trees respond to elevated O_3_ exposure in situ and how well the trees recover in terms of BVOC emissions and photosynthetic rate after exposure. How mature birch trees respond to elevated O_3_ at different leaf ages is also uncertain and can be of importance as the stress response can appear differently depending on leaf age, and a high O_3_ level early in the growing season may reduce photosynthetic capacity at an early stage. To enhance our understanding of the responses of mature mountain birch trees in sub‐Arctic areas to exposure to elevated O_3_, we defined the following aims: (1) investigate the effect of leaf age on BVOC emissions and photosynthetic rate, and (2) compare the potential difference in acute O_3_ exposure effects on young leaves and older leaves. We also aimed to (3) quantify the effect of different acute O_3_ exposure levels to identify thresholds in plant defense for carbon assimilation and BVOC emission, and (4) analyze the recovery of birch leaves after exposure to acutely increased O_3_ concentrations.

## 2 METHODS

### 2.1 Site description

Field measurements were done during the growing season of 2020 in the nature reserve at the Abisko Scientific Research Station (ANS) in the Swedish sub‐Arctic (68°20′ N 19°02′ E) inside a birch forest dominated by mountain birch (*Betula pubescens* ssp. *czerepanovii*). The field site was visited twice, during the start of summer (22nd of June–3rd of July) and during the peak growing season (27th of July–5th of August), herein referred to as early and late summer (Table 1). All trees in the area had started to develop leaves about 2 weeks before the first field visit (8th of June; ANS, 2023). The leaves were fully unfolded when the measurement started. The area has a history of autumnal moth (*Epirrita autumna*) outbreaks every 10–11 years that strongly modify the BVOC emissions of mountain birch when numerous larvae eat the leaves (Rieksta et al., 2020). There was no indication of an outbreak during the measurement year and the latest outbreak occurred in 2012/2013 (Olsson et al., 2017; Tenow, 1996), meaning that the measured emission rates during the study were not influenced by herbivory.

**TABLE 1:** Sampling conducted during 2020 at the field site in Abisko (northern Sweden) for the field visits early and late in the summer.

Date	Number of measured leaves and collected samples for each tree							
	Birch 1		Birch 2		Birch 3		Total	
	Leaves	Samples	Leaves	Samples	Leaves	Samples	Leaves	Samples
28th of June to 4th of July	3	22	6	25	3	21	12	68
28th of July to 3rd of August	6	30	3	21	4	28	13	79
Total							25	147

*Note*: The same three birch trees and branches were measured during both field visits, and the number of leaves measured each time along with the number of samples and the total amount of leaves and samples were summed for each field visit and the entire study.

**FIGURE 1:**
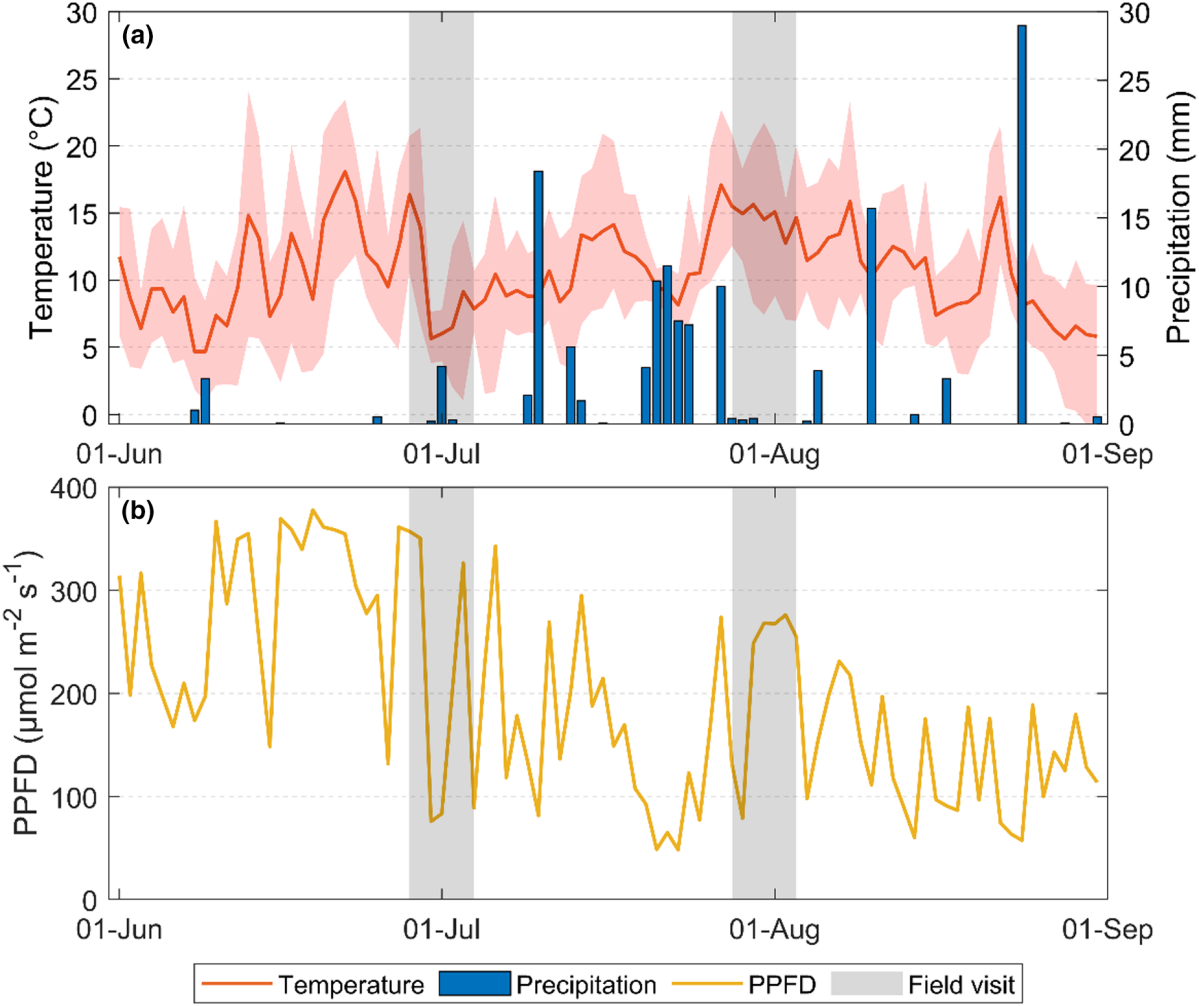
The measured (a) daily average air temperature (red line with daily maximum and minimum in a red‐shaded area) and daily precipitation (blue bar) and (b) daily average incoming photosynthetic active radiation (PPFD; yellow line) for the growing season of 2020 at the Abisko Scientific Research Station. The gray‐shaded areas indicate the time of the field visits for this study. Temperature and precipitation data were supplied by the Swedish Meteorological and Hydrological Institute (SMHI, 2023), and radiation data were supplied through the Abisko Scientific Research Station (ANS, 2023).

The long‐term mean air temperature (year 1991–2020) during the growing season (June to August) in Abisko ranges from 9°C to 12°C with a precipitation sum of 140 mm (SMHI, 2023). In 2020, the growing season was a bit warmer than the long‐term average (15 ± 3°C (mean ± standard deviation)), while the precipitation was similar to the long‐term average with 143 mm. The daily amplitude in temperature was large; the lowest and highest temperatures during the first field visit were 1°C and 21°C, while it was slightly warmer during the later field visit, with the lowest and highest temperatures being 6°C and 22°C (Figure 1). The sum of precipitation during the field visits was 5 mm and 1 mm, respectively.

Three mature mountain birch trees growing within 4 meters of each other were selected for repeated measurements during the study period (Table 1). The trees were at least 40 years old and between 3 and 4 m tall. One branch from each tree was selected for the study; the branches on birch 1 and 2 were at similar heights of around 1.40 m, and the branch on birch 3 was located further down the tree at around 0.7 m. Leaves in direct sunlight were excluded from the selection. The same branches were measured during both field visits.

### 2.2 Experimental design

**FIGURE 2:**
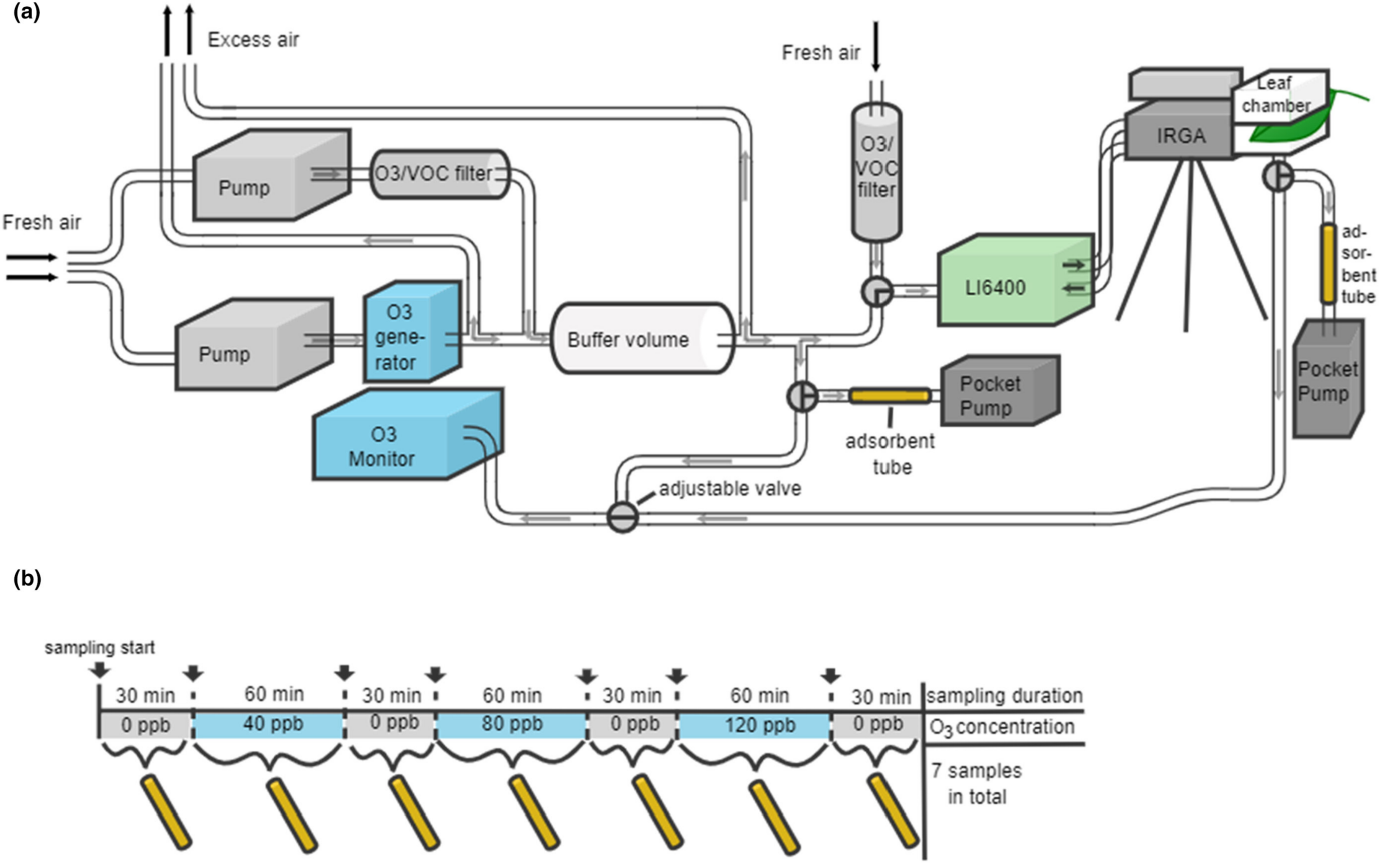
Schematic of the (a) instrumental set‐up and (b) measurement sequence. Ambient air entered the system from three inlets, one through a VOC filter and one through an O_3_ generator, which were later joined in a buffer volume to deliver O_3_‐enriched air to the system. The third air inlet was located directly at the inlet of the photosynthesis system (LI6400) and included a scrubber for VOCs and O_3_ to use as O_3_‐free air during the 0 ppb measurements. During the O_3_ exposure measurements, the O_3_‐enriched air was directed to an O_3_ monitor and the LI6400. Air was sampled through adsorbent tubes using pocket pumps from two places: after the chamber head (for leaf emissions) and after the buffer volume (to get background emissions). Using an automatic toggling three‐way valve, the O_3_ monitor was receiving air before the LI6400 and after the chamber head to see concentrations before and after the chamber. One leaf was sampled for 30 min at 0 ppb before O_3_ exposure, after which it was exposed to 40 ppb, 80 ppb, and 120 ppb for 60 min each, with a recovery phase of 30 min in‐between. Samples were taken at each step in the sequence, resulting in seven samples in total for one leaf.

The measurement setup consisted of a pump system and a portable photosynthesis system (LI‐6400; LICOR, Lincoln, NE, USA), combined with an O_3_ monitor (Model 202; 2B Technologies, Boulder, CO, USA) and an O_3_ generator (Certizon C25; Erwin Sander Elektroapparatebau GmbH, Am Osterberg, Germany) connected with PTFE tubing (∅ 6.35 mm; Teflon, Swagelok, Solon, OH, USA). A schematic of the instrument setup, connections, and inlets and outlets are shown in Figure 2a. The pump provided ambient air to the system, passing the O_3_ generator that was manually adjusted to different concentration levels (i.e. 40, 80, or 120 ppb). The O_3_‐enriched air was passed through a buffer volume of ~3 liters mixed with filtered 0 ppb O_3_ air to enable stable concentrations. From the buffer volume, the air was led through a T‐cross to both the LI‐6400 leaf chamber and the O_3_ monitor that was used to measure the O_3_ concentration reaching the leaf chamber. An adjustable T‐cross was also installed before the photosynthesis system for swapping between O_3_‐enriched air and filtered 0 ppb O_3_ air. When the 0 ppb O_3_ measurements were ongoing, the T‐crossing was directed to only have filtered 0 ppb O_3_ air entering the chamber. When the O_3_ exposure measurements started, this T‐crossing was directed toward the O_3_‐enriched air. Once the air passed the chamber head, it was directed to the O_3_ monitor to assess the O_3_ concentration the leaf was experiencing. From the same air stream, BVOC samples were taken by extracting air through adsorbent tubes using a pocket pump (SKC Ltd., Dorset, UK). During the sampling period, there was an automatic toggling three‐way valve installed to swap between air before and after the chamber with 2‐min intervals to monitor the different O_3_ levels and potential degradation through the photosynthesis system to know the approximate concentration inside the leaf chamber. Each leaf was measured at all O_3_ concentration steps following a measurement sequence starting at 0 ppb (control) for 30 min, followed by an exposure phase of 40 ppb for an hour, back to 0 ppb for 30 min again (recovery phase 1), an exposure phase of 80 ppb for an hour, 0 ppb for 30 min (recovery phase 2), 120 ppb for an hour, and, lastly, 0 ppb for 30 min (recovery phase 3; Figure 2b). Each leaf was sampled for BVOC seven times following the sequence (Figure 2b), resulting in a total of 147 samples (Table 1). After each measurement sequence, the leaves were harvested and photographed for specific leaf area (SLA) calculations. They were weighted and dried at 70°C until the dry weight was stable. Additional measurements of chlorophyll content were taken on five leaves on the same branch using a chlorophyll meter (SPAD‐502 Plus, Konica Minolta Sensing, Inc., Japan).

### 2.3 BVOC sampling

Before starting the measurements, the leaves were inserted into the chamber of the LI‐6400 with a flow rate of 750 μmol s^−1^ and allowed an hour to acclimate to a leaf temperature of 20°C and photosynthetically active radiation (PAR) of 1000 μmol m^−2^ s^−1^. The CO_2_ concentration during the measurements varied with the ambient concentration, ranging from 350 to 430 ppm. BVOCs were sampled by drawing air through adsorbent tubes (Markes International Limited, Llantrisant, UK) packed with Tenax TA (a porous organic polymer) and Carbograph 1TD (graphitized carbon black) using flow‐controlled pocket pumps (Pocket Pump; SKC Ltd., Dorset, UK). To avoid O_3_ oxidation of the BVOCs inside the tube, an O_3_ filter (MnO_2_ nets) was installed in front of the tube. The sampling time during the exposure phases was 60 min with a flow rate of 100 mL min^−1^ through the tubes, while the sampling time was 30 min with a flow rate of 200 mL min^−1^ during the control and recovery stages. The collected volume for each sample was 5 to 6 L. The net photosynthetic rate was measured simultaneously with the BVOC sampling using the LI‐6400, herein only referred to as the photosynthetic rate.

To identify possible background contamination, background BVOC concentrations were sampled through similar adsorbent tubes and pocket pumps at two locations in the experimental setup: after the buffer volume and directly after the chamber with no leaf inside (Figure 2a). The background samples were done twice a day and sampled for 30 min with a flow rate of 200 mL min^−1^. After collecting the samples, all tubes were capped with long‐term storage caps and stored in a refrigerator (~3°C) before analysis.

### 2.4 BVOC analysis

The adsorbent tubes were analyzed using a thermal desorption unit (TD; TurboMatrix 350, Perkin‐Elmer) connected to a gas chromatograph (GC; Clarus 680, Perkin‐Elmer) coupled to a mass spectrometer (MS; Clarus SQ 8 T, Perkin‐Elmer). The samples were cryo‐focused onto a dual‐adsorbent cold trap (Tenax TA and Carbopack B) kept at −30°C. A DB‐5 column (length 60 m, internal diameter (id.) 0.25 mm, film thickness 1 μm, from Agilent Technologies) was used for separation. TD‐GC–MS was calibrated using methanol solutions of BVOCs injected into the sorbent tubes using nitrogen (99.9999%) with a flow of ~80 mL min^−1^. Isoprene was calibrated with a gaseous standard from National Physical Laboratories (NPL, UK). Quantified compounds included isoprene, 6 MTs (α‐pinene, β‐pinene, camphene, 3Δ‐carene, p‐cymene, and limonene), 5 SQTs (longicyclene, iso‐longifolene, β‐caryophyllene, β‐farnesene, α‐humulene), and 5 oxygenated‐BVOCs (OXY; 2‐methyl‐3‐buten‐1ol (MBO), cis‐3‐hexenol, linalool, 4‐acetyl‐1‐methylcyclohexene (AMCH), nopinone). The method has been described in Helin et al. (2020).

The leaf dry weight (g_dw_) inside the chamber was calculated according to Equation (1):

(1) $$g_{\mathrm{dw}}=\frac{g_{\mathrm{dw}\;\mathrm{tot}}}{\mathrm{LA}}\times \mathrm{CA},$$

where g_dw tot_ is the dry leaf weight of the whole leaf (g), LA is the total leaf area (cm^2^), and CA is the leaf area inside the chamber, which was always 6 cm^2^.

The analyzed BVOC concentrations were calculated as emission rates (ER; ng g_dw_^−1^ h^−1^) following Ortega and Helmig (2008). As PAR and leaf temperature were set to constant values, the emission rates were not standardized but reported as measured emission rates at 20°C and 1000 μmol m^−2^ s^−1^ PAR. The compound blend (%) was calculated as the emission of the emitted compounds divided by the sum of the emissions of all identified BVOCs. The ratio between compound groups were calculated similarly. The detection limit (LOD) was calculated for each compound as emission rates based on blank measurements resulting in LOD ranging from 0.45 to 10 ng g_dw_^−1^ h^−1^ (see Table S2 for LOD for each compound).

SLA (cm^2^ g^−1^) was calculated as LA divided by g_dw tot_.

### 2.5 Statistical analysis

Our data sets were tested for normality using the Kolmogorov–Smirnov test and creating normal probability plots (kstest, normplot, MATLAB 2022b, Mathworks, Inc., MA, USA), resulting in no normal distribution of the data. Any significant differences between the control leaves from the early and late summer were tested for leaf photosynthetic rate, stomatal conductance, transpiration rate, SLA, chlorophyll content, and the BVOC emission rates for unique compounds and the total emissions with a Kruskal–Wallis test (kruskalwallis, MATLAB R2022b; MathWorks, Inc., MA, USA) with a significance level set to *p* < .05. The significant differences for all leaves during the early or late summer between each step in the measurement sequence were also tested for photosynthetic rate, stomatal conductance, and the BVOC emission rates for unique compounds and total BVOC emissions, using two separate Kruskal–Wallis tests for each measurement time (early or late summer). After the Kruskal–Wallis test, a multiple comparisons procedure with Dunn–Sidák's approach (multcompare, MATLAB R2022b; MathWorks, Inc., MA, USA) was made to identify which groups were significantly different from each other, if any. Rough winds broke off some leaves during sampling; these leaves were removed from the data set and not analyzed.

## 3 RESULTS

### 3.1 BVOC emission patterns and photosynthetic rate from non‐exposed (control) birch leaves

**FIGURE 3:**
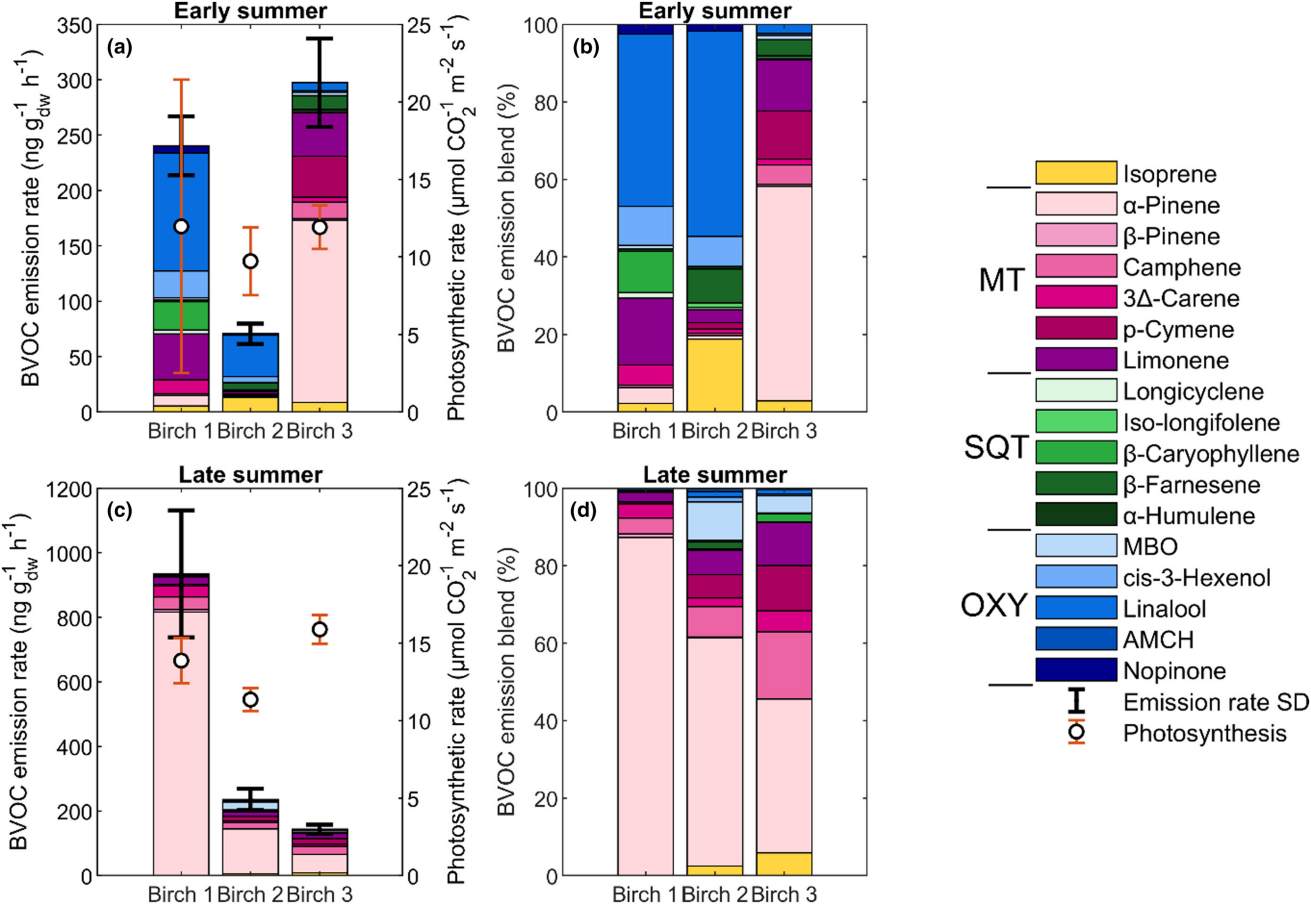
The non‐exposed (control) birch branches: (a) early summer BVOC emission (mean ± standard deviation (SD)) and photosynthetic rate (mean ± SD), (b) early summer emission blend (%), (c) late summer BVOC emission (mean ± SD) and photosynthetic rate (mean ± SD), and (d) late summer emission blend (%). The BVOC compounds are separated by color in their respective groups, with monoterpenes (MT) in red‐pink, sesquiterpenes (SQT) in greens, and oxygenated compounds (OXY) in blues, where MBO is abbreviated from 2‐methyl‐3‐buten‐1‐ol and AMCH from 4‐acetyl‐1‐methylcyclohexene. Isoprene is separated on its own and colored yellow.

The first aim was to study differences depending on leaf age. Following the O_3_ exposure sequence, mountain birch leaves (*Betula pubescens* ssp. *czerepanovii*) were measured starting at a concentration of 0 ppb to measure the constitutive BVOC emissions. The results show that early leaves had an average BVOC emission rate of 189 ± 189 ng g_dw_^−1^ h^−1^ (mean ± standard deviation) for the total BVOC compounds, while the late leaves emission rate was higher with an average of 456 ± 876 ng g_dw_^−1^ h^−1^ (mean ± standard deviation; Figure 3; Table S2). Separated into compound groups, early leaf MT emission was 100 ± 170 ng g_dw_^−1^ h^−1^ and late leaf MT emission was higher at 433 ± 882 ng g_dw_^−1^ h^−1^. Oxygenated compounds (OXY), SQT, and isoprene emissions were instead higher for the early leaves (OXY: 21 ± 51, ng g_dw_^−1^ h^−1^ SQT: 17 ± 25 ng g_dw_^−1^ h^−1^, isoprene: 10 ± 16 ng g_dw_^−1^ h^−1^) compared to the late leaves (OXY: 5 ± 13 ng g_dw_^−1^ h^−1^, SQT: 4 ± 4 ng g_dw_^−1^ h^−1^, isoprene: 5 ± 8 ng g_dw_^−1^ h^−1^). The early leaf photosynthetic rate was on average 11 ± 5 μmol m^−2^ s^−1^ and 14 ± 2 μmol m^−2^ s^−1^ for the late leaves (Table S2), which shows a relatively higher fraction of resources spent in spring for OXY, SQT, and isoprene, but the opposite for MT. Despite the higher total emission rates for the late leaves, the difference was not significant. However, the emission rates for two individual compounds indicated a significant difference: camphene was significantly higher (*p* < .03) for the late leaves, and longicyclene was significantly lower (*p* < .003) for the late leaves compared to the early leaves (Table S2). The photosynthetic rate was significantly higher for the late leaves (*p* < .03; Table S2), along with significant differences for SLA, chlorophyll content, transpiration rate, and stomatal conductance (*p <* .001 for all), where SLA was higher for early leaves but chlorophyll content, transpiration rate, and stomatal conductance were lower compared to late leaves (Table S2).

We found high variabilities in BVOC emission rates between leaves from different branches and also among leaves from the same branch (Figure 3; Figure S2). The compound blend also varied much, especially for the early leaves, where half emitted mainly OXY compounds (Table S1). The emissions from late leaves were mainly dominated by α‐pinene for all but two leaves (Table S1).

### 3.2 BVOC emission patterns and photosynthetic rate during the recovery phase

**FIGURE 4:**
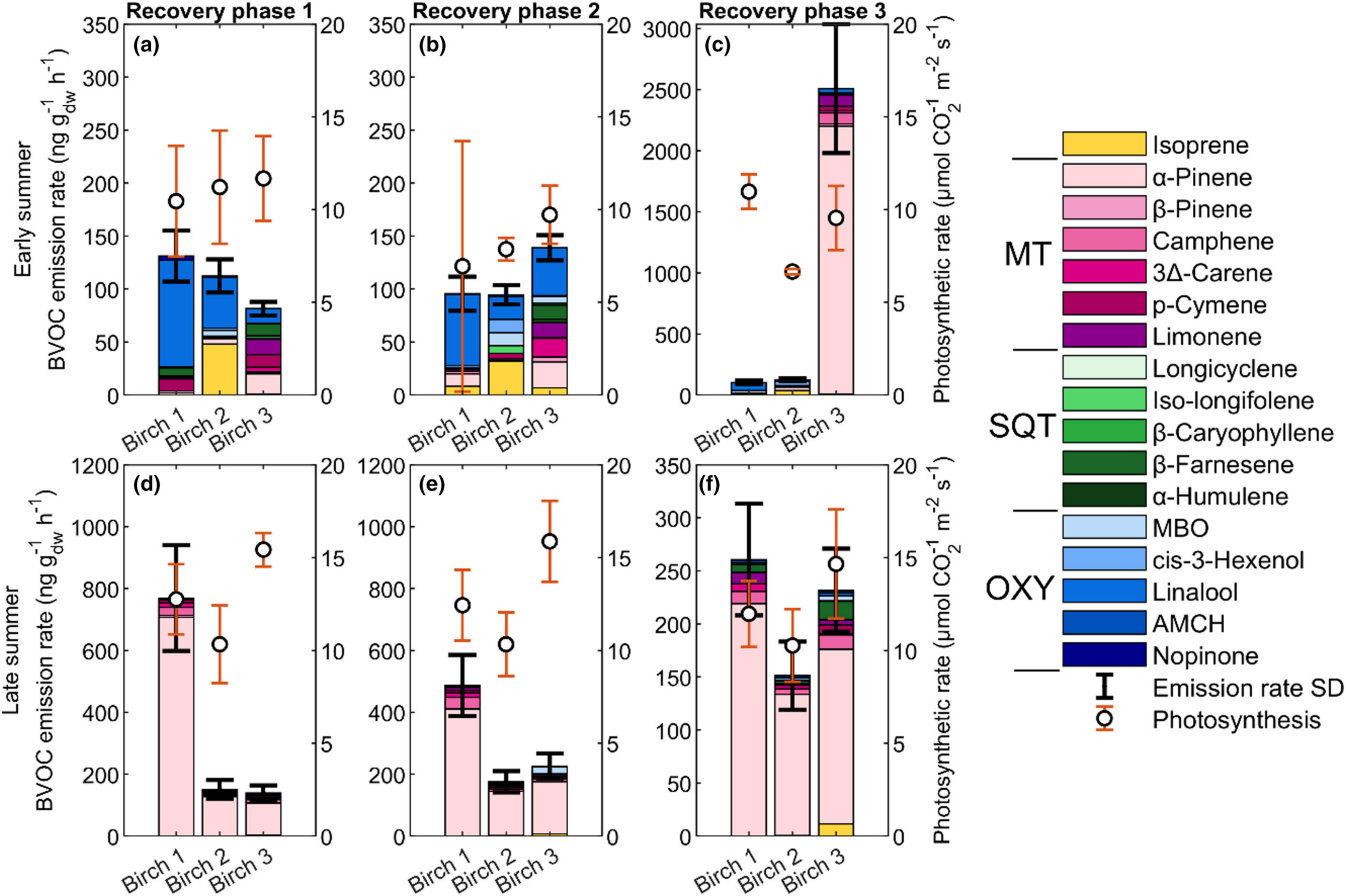
The recovery‐phase birch branches' (a) early summer BVOC emission and photosynthetic rate during recovery phase 1, (b) early summer BVOC emission and photosynthetic rate during recovery phase 2, (c) early summer BVOC emission and photosynthetic rate during recovery phase 3, (d) late summer BVOC emission and photosynthetic rate during recovery phase 1, (e) late summer BVOC emission and photosynthetic rate during recovery phase 2, and (f) late summer BVOC emission and photosynthetic rare during recovery phase 3. The birch leaves were exposed to elevated O_3_ at concentrations of 40 ppb, 80 ppb and 120 ppb prior to measuring the respective recovery phases 1–3 at an exposure concentration of 0 ppb. The standard deviation (SD) from the mean for the BVOC emission rates is given by the black whiskers. The BVOC compounds are separated by color in their respective groups, with monoterpenes (MT) in red‐pink, sesquiterpenes (SQT) in greens, and oxygenated compounds (OXY) in blues, where MBO is abbreviated from 2‐methyl‐3‐buten‐1‐ol and AMCH from 4‐acetyl‐1‐methylcyclohexene. Isoprene is separated on its own and colored yellow.

One of the aims was to investigate the extent to which the trees recovered after short‐term O_3_ exposure. The measurement sequence included three recovery phases where the leaves were exposed to 0 ppb after exposure to 40 ppb, 80 ppb, and 120 ppb O_3_ concentrations. The birch trees showed different recovery patterns. Total emission rates from Birch 1 decreased for all recovery phases compared to the control for both early and late leaves. Birch 2 increased emission rates with each recovery phase for early leaves, while the emission rates for late leaves decreased during the recovery phases compared to the control state. The early leaves from Birch 3 indicated decreased emission rates for the first two recovery phases compared to the control, but increased to five times higher emission rates at the last recovery phase. The late leaves instead indicated small increases in emission rates during the recovery phases (Figure 4; Table S3). The emission blend for late leaves was similar to the control, mainly the emission of α‐pinene for all birch branches and recovery phases (Figure S3). However, the recovery phases for the early leaves varied among the birches, with main increases in compound contribution (% of total emissions) for linalool (50%), isoprene (20%), MBO (15%), and cis‐3‐hexenol (8%) on average for Birch 1 and 2. Birch 3, on the other hand, had the largest increase both in emission rate and emission contribution, dominated by α‐pinene with 87% during the third recovery phase (Figure 4; Figure S3).

Similar photosynthetic rates were observed for early leaves between the birches during the control state and the first recovery phase (10–12 μmol m^−2^ s^−1^; Figures 3 and 4). Photosynthetic rates did, however, decrease by 3 μmol m^−2^ s^−1^ for all birches during the second recovery phase compared to the first (Figure 4). However, during the third recovery, they responded differently in photosynthetic rate: Birch 1 increased to similar rates as the control and first recovery phase (11 μmol m^−2^ s^−1^), Birch 2 continued to decrease in photosynthetic rate to 7 μmol m^−2^ s^−1^, and Birch 3 showed no difference from the second recovery phase (Figure 4). The late leaves instead have a larger difference in photosynthetic rate between the birches in the control state, 11, 13, and 15 μmol m^−2^ s^−1^ for Birches 1, 2, and 3, respectively. However, the photosynthetic rate instead remained stable throughout all recovery phases for all birches (Figure 4).

### 3.3 BVOC emission patterns and photosynthetic rate during O_3_ exposure

**FIGURE 5:**
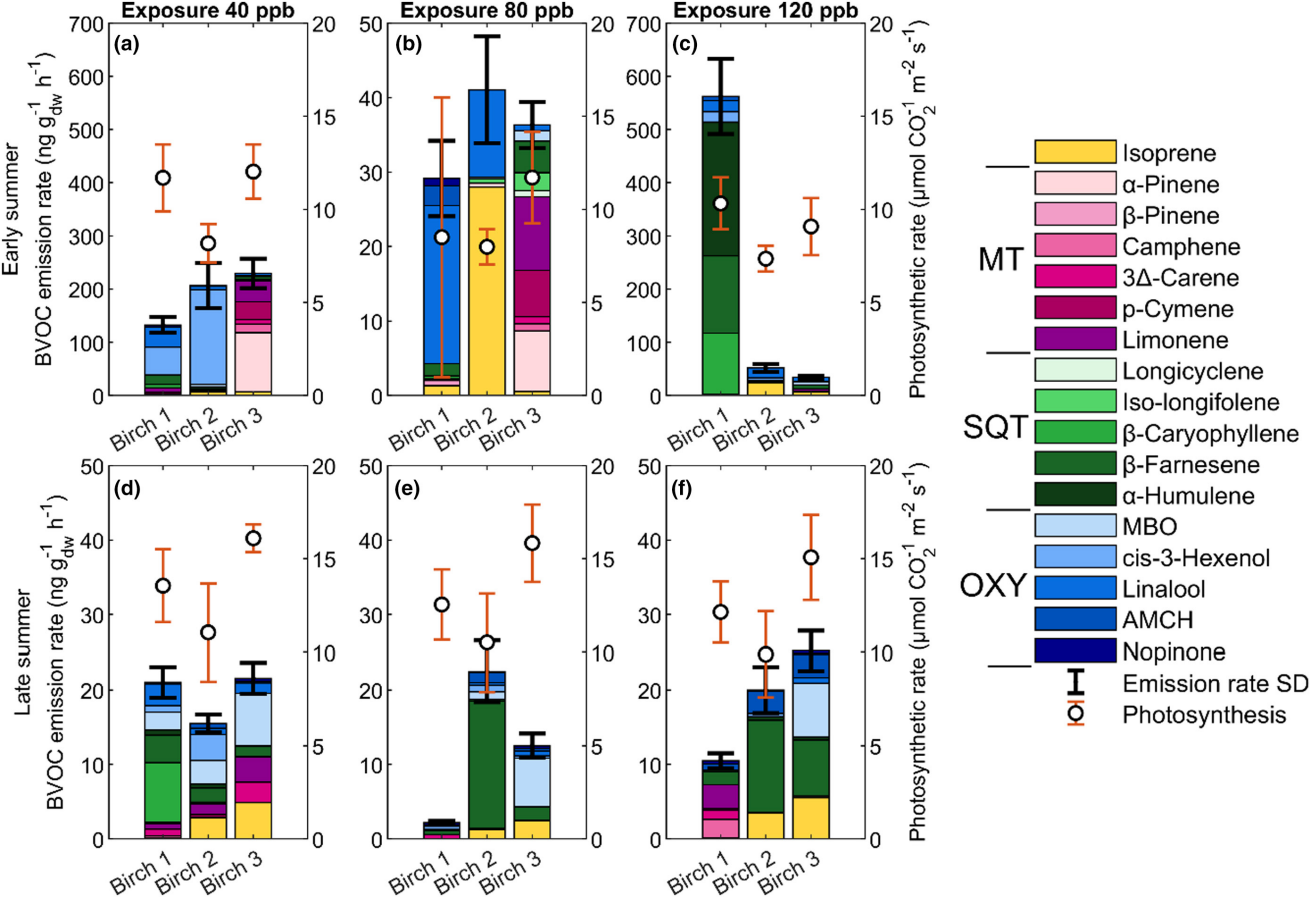
The O_3_ exposure‐phase birch branches' (a) early summer BVOC emission and photosynthetic rate at 40 ppb, (b) early summer BVOC emission and photosynthetic rate at 80 ppb, (c) early summer BVOC emission and photosynthetic rate at 120 ppb, (d) late summer BVOC emission and photosynthetic rate at 40 ppb, (e) late summer BVOC emission and photosynthetic rate at 80 ppb, and (f) late summer BVOC emission and photosynthetic rare at 120 ppb. The birch leaves were exposed to elevated O_3_ at concentrations of 40 ppb, 80 ppb, and 120 ppb following a recovery phase of 0 ppb. The standard deviation (SD) from the mean for the BVOC emission rates is given by the black whiskers. The BVOC compounds are separated by color in their respective groups with monoterpenes (MT) in red‐pink, sesquiterpenes (SQT) in greens, and oxygenated compounds (OXY) in blues, where MBO is abbreviated from 2‐methyl‐3‐buten‐1‐ol and AMCH from 4‐acetyl‐1‐methylcyclohexene. Isoprene is separated on its own and colored yellow.

To investigate the effects of O_3_ exposure on leaf age and find a potential stress threshold, the exposure phases of the measurement sequence included step‐wise increases in the O_3_ concentration, which can be considered a light (40 ppb), medium (80 ppb), and severe (120 ppb) O_3_ stress level. We found that, similar to the control and recovery stages, the emission blend profile varied considerably for both early and late leaves (Figure S4). The emission rates depending on leaf age indicated differences during the different exposure stages. Total emission rates were 2–50 times higher from early leaves compared to late leaves during the same exposures of 40 ppb and 120 ppb (Figure 5; Table S4). The differences between early and late leaves were slightly smaller at 80 ppb, but emission rates were still 2 times higher for the early leaves (Figure 5; Table S4).

The early leaves from Birches 1 and 2 had similar patterns where their emission blend indicated a 95% increase of cis‐3‐hexenol when exposed to 40 ppb compared to the control, while Birch 3 was deviating from the pattern with an 80% increase of α‐pinene instead (Figure S4). Compared to the control, the early leaves from Birch 1 and 3 decreased the total emission rates with 112 and 68 ng g_dw_^−1^ h^−1^ at 40 ppb, respectively, while Birch 2 instead increased emission rates with 129 ng g_dw_^−1^ h^−1^. At 80 ppb, the compound blend of Birch 2 was dominated by linalool (70%) and Birch 3 by isoprene (70%), while Birch 1 had limonene (30%) and α‐pinene (20%) as main contributors (Figure S4). Emission rates were 80% lower for all birches at 80 ppb compared to 40 ppb and control (Figure 5; Table S4). Exposure at 120 ppb revealed no large change in emission rates or compound blend for Birch 2, other than small (>8%) compound blend increases in cis‐3‐hexenol and MBO compared to 80 ppb (Figure S4; Figure 5; Table S4). Exposure at 120 ppb for Birches 1 and 3 had larger implications in their blend. SQTs α‐humulene, β‐caryophyllene, and β‐farnesene dominated the emissions (90%) from Birch 1 that also had the highest emission rates (560 ng g_dw_^−1^ h^−1^) of all stages (Figure S4; Figure 5; Table S4). Birch 3 mainly emitted MBO, linalool, isoprene and β‐farnesene (together 77% of their emissions), with emission rates remaining similar to the exposure at 80 ppb (Figure S4; Figure 5; Table S4). The photosynthetic rates were seen to decrease on average by 15% and 19% at each step for Birches 1 and 2, respectively, compared to the control stage, while they were stable for Birch 3 until the last exposure stage (120 ppb) where they decreased (Figure 5; Table S4).

When the late leaves were exposed to 40 ppb, all emissions changed from α‐pinene dominance (40%–80% of total emissions) to a higher variety of mainly MBO, limonene, isoprene, and β‐caryophyllene (Figure S4). The emission rates for all control stage late leaves were 40 times higher than the late leaf emission rates at 40 ppb (Table S4). When the leaves were exposed to 80 ppb, larger differences in emission rates and compound blends were seen. Birch 1 decreased further in emission rate to 2 ng g_dw_^−1^ h^−1^ and changed the blend to an even distribution of nopinone, cis‐3‐hexenol, 3∆‐carene, β‐farnesene, and AMCH (Table S4; Figure 5; Figure S4). Birch 2 also changed in the compound blend to mainly β‐farnesene (76%), but slightly increased emission rates compared to 40 ppb (15 to 22 ng g_dw_^−1^ h^−1^; Table S4; Figure 5; Figure S4). Similarly, Birch 3 changed its blend to be MBO‐dominated (50%) with a decrease in emission rates compared to 40 ppb (22–12 ng g_dw_^−1^ h^−1^; Figure S4; Figure 5; Table S4). At 120 ppb, Birch 2 and 3 increased AMCH (13%) and β‐farnesene (62% and 30%, respectively) in their blend, while Birch 1 additionally increased in limonene (30%) and camphene (20%; Figure S4). The emission rates at 120 ppb were higher for Birches 1 and 3 compared to 80 ppb (8 ng g_dw_^−1^ h^−1^ and 13 ng g_dw_^−1^ h^−1^ higher, respectively), while they remained at similar levels for Birch 2 (20 ng g_dw_^−1^ h^−1^; Figure 5; Table S4). The photosynthetic rates remained at similar levels throughout the exposure sequence for all birches (Birch 1: 13 μmol m^−2^ s^−1^, Birch 2: 10 μmol m^−2^ s^−1^, and Birch 3: 16 μmol m^−2^ s^−1^; Figure 5; Table S4).

### 3.4 The average BVOC emission and photosynthetic rate for all measurement sequence steps

**FIGURE 6:**
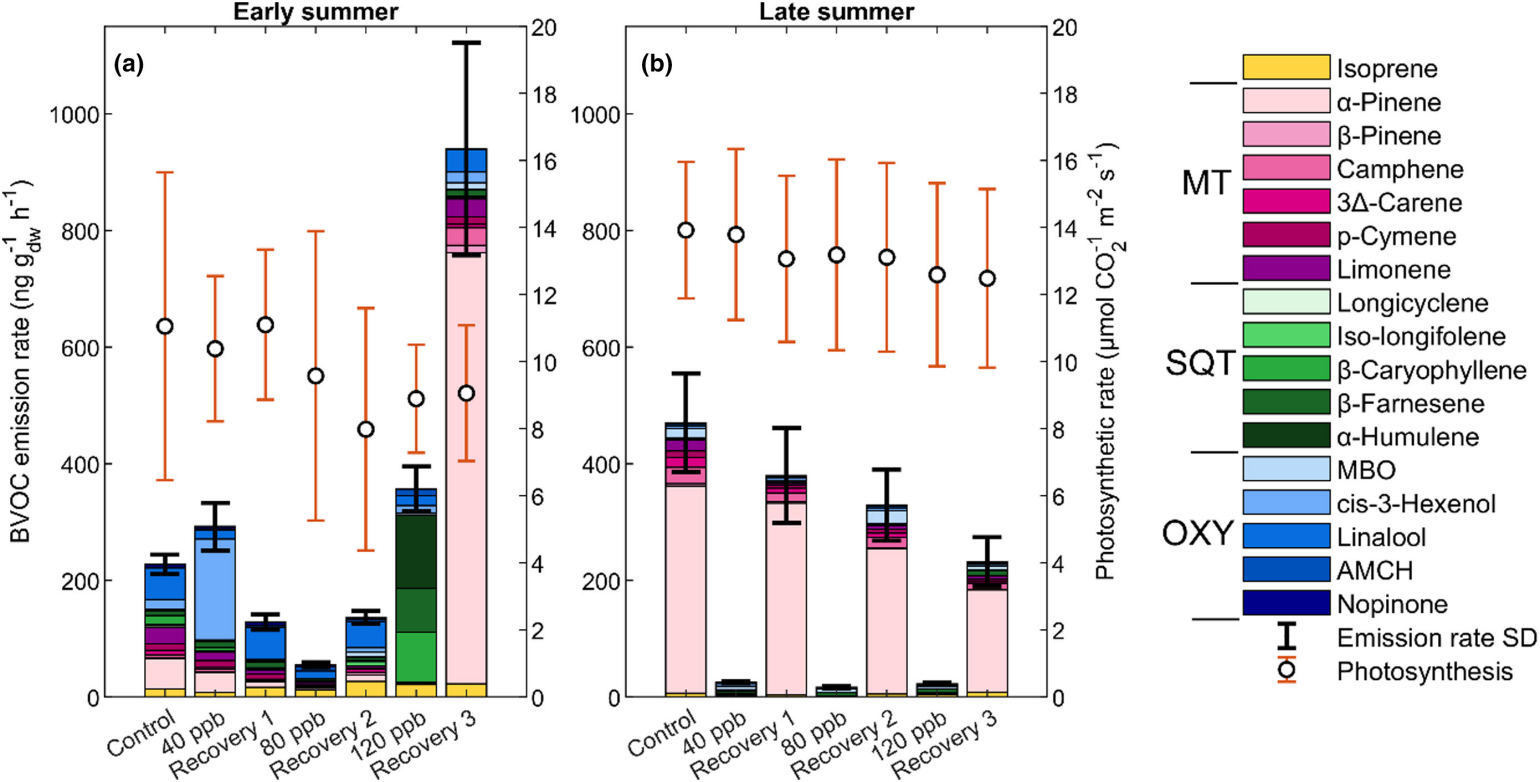
The average BVOC emission and photosynthetic rate for each step in the measurement sequence (control state, exposed to 40 ppb, recovery 1 (0 ppb), exposed to 80 ppb, recovery 2, exposed to 120 ppb, recovery 3) for (a) early summer and (b) late summer. The standard deviation from the mean (SD) for the BVOC emission rates is given by the black whiskers. The BVOC compounds are separated by color in their respective groups, with monoterpenes (MT) in red‐pink, sesquiterpenes (SQT) in greens, and oxygenated compounds (OXY) in blues, where MBO is abbreviated from 2‐methyl‐3‐buten‐1‐ol and AMCH from 4‐acetyl‐1‐methylcyclohexene. Isoprene is separated on its own and colored yellow.

**FIGURE 7:**
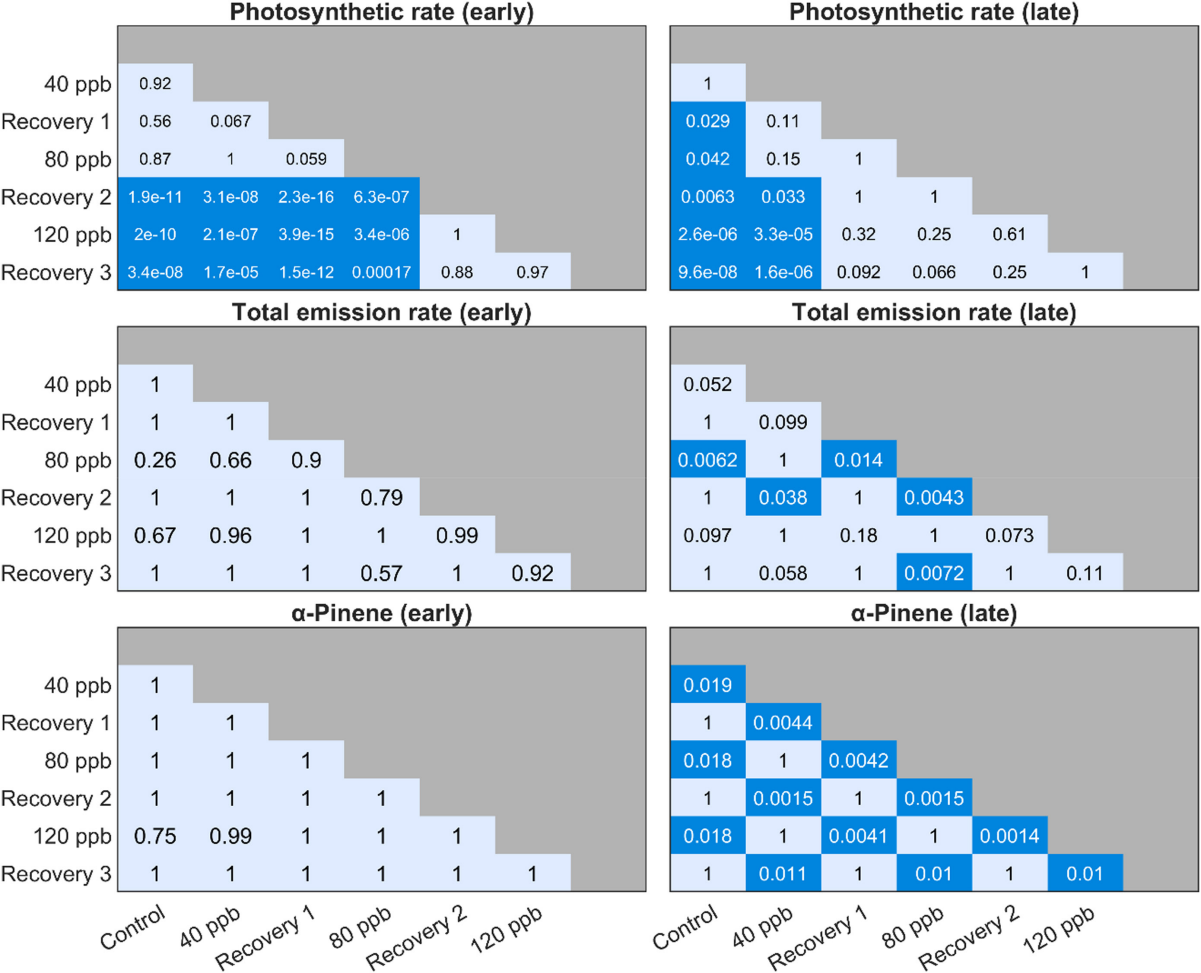
The resulting *p*‐values between the steps in the measurement sequence for both early and late summer photosynthetic rates, total emission rates, and from the compound α‐pinene. A dark blue box indicates a significant difference (*p* < .05) between the control/exposure/recovery phases. Statistical analysis was performed using a Kruskal–Wallis test followed by a multiple comparisons procedure with Dunn–Sidák's approach to investigate which groups were different from each other.

An average of the BVOC emission and photosynthetic rate was calculated for early and late leaves. The average followed similar patterns as described for the individual birches; early leaves had no large difference between control and 40 ppb, only increases of cis‐3‐hexenol (16 to 73 ng g_dw_^−1^ h^−1^; Table S4; Figure 6a). The early leaves had non‐significantly lower emission rates compared to the control at recovery 1, 2, and 80 ppb, but the emission rates non‐significantly increased with 139 ng g_dw_^−1^ h^−1^ at 120 ppb and changed in blend to mainly SQTs (80%; Figure 6a). At recovery 3, the early leaf emission rate non‐significantly increased 5 times compared to the control state, where α‐pinene contributed 80% to the increase (Figure 6a). The late leaves had a more obvious pattern, with significantly lower (*p* < .02) total emission rates at all exposure stages compared to the control and recovery stages (Figure 6b; Figure 7). The recovery stages also indicated a steady but non‐significant decrease in emission rate after each exposure, where the emissions decreased on average by 20% after exposure compared to the control stage (Figure 6b).

The photosynthetic rate showed tendencies to decrease at each step compared to the control state, irrespective of leaf age, where the decrease from control to last recovery was 2 μmol m^−2^ s^−1^ for early leaves and 1.5 μmol m^−2^ s^−1^ for late leaves (Figure 6a; Table S3; Table S4). Stomatal conductance for early and late leaves followed similar patterns, where the main difference between them was the magnitude, as the late leaves had higher stomatal conductance compared to the early leaves (10 mol H_2_O m^−2^ s^−1^ higher; Figure S1). After exposure at 80 ppb, there was always a significant decrease in the early leaf photosynthetic rate compared to the control leaves at 40 ppb and recovery 1 (*p <* .001; Figure S5). The stomatal conductance of the early leaves was significantly lower during exposure to 80 ppb compared to the control (*p* < .001; Figure S5). Despite this, there was no significant difference in the total emission rates or the compounds for the early leaves (Figure S5). The late leaves, however, had a significantly lower photosynthetic rate already after 40 ppb compared to the control (*p* < .03), but only significantly lower stomatal conductance after exposure to 120 ppb compared to the control (*p* < .001; Figure S6). Late leaves also had significantly lower emissions of α‐pinene (*p* < .02) when the leaves were exposed to O_3_ (Figure 7). There were also small differences for the compounds β‐caryophyllene, p‐cymene, 3Δ‐carene, and camphene, which had the main differences between the control state and the exposure phases (*p* < .05; Figure S6). Only camphene had significant differences between recovery 1 and 80 ppb (*p* < .05; Figure S6).

### 3.5 Changes in the ratio of monoterpenes to sesquiterpenes depending on exposure and recovery

**FIGURE 8:**
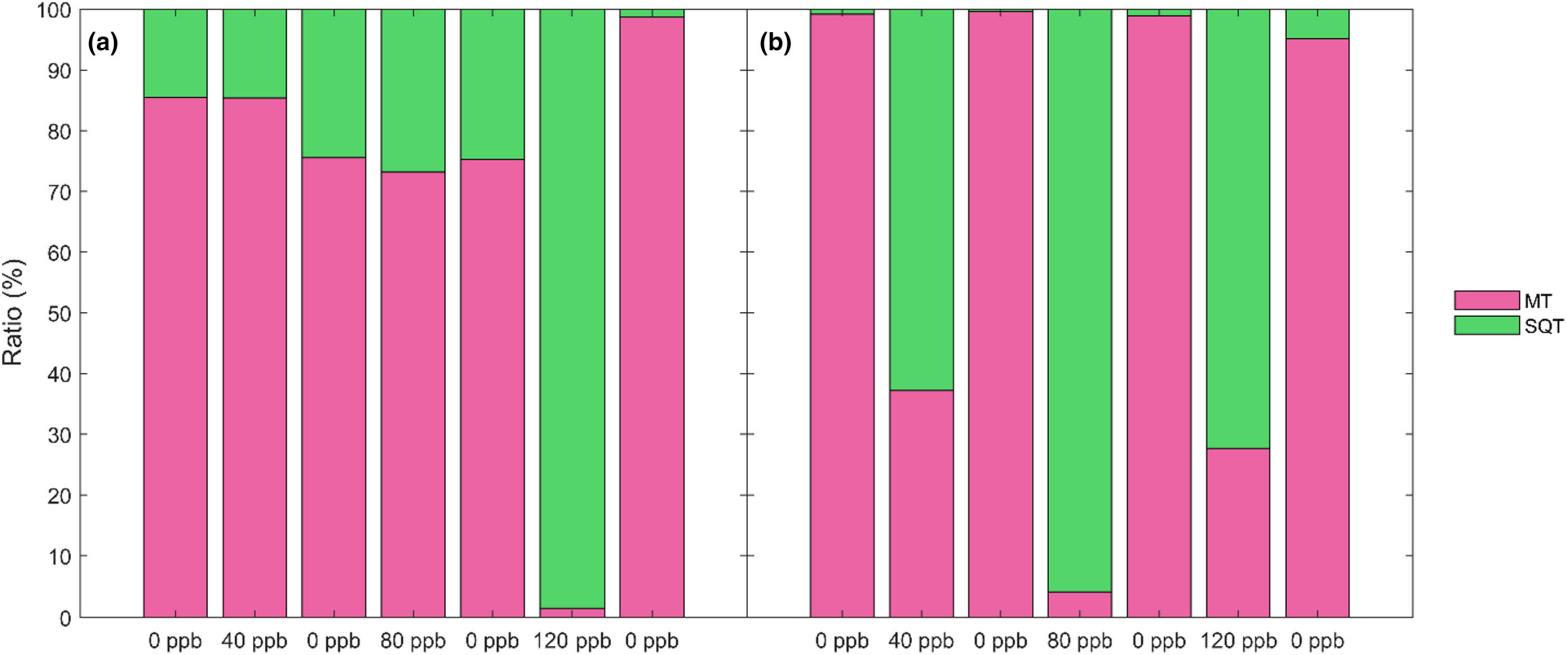
The emission rate ratio in percent (%) for (a) early summer and (b) late summer emissions of the group monoterpenes (MT) and the group sesquiterpenes (SQT). The ratio between the BVOC compound groups is displayed for each step of the measurement sequence (non‐exposed, exposed to 40 ppb, 80 ppb, and 120 ppb and the respective recovery phases).

As a further stress indicator, the ratio between MT and SQT was relevant due to their different production pathways. When comparing the sum of the MT and SQT for early leaves, we saw that MT dominated all steps except at 120 ppb, where SQT was dominating at almost 100%, but the opposite was seen at recovery 3 (Figure 8a). For the late leaves, there was a clear division between the ratios for the control and recovery stages compared to the O_3_ exposure stages: MT was always dominating when there was 0 ppb O_3_, while SQT was dominating during the exposure stages (Figure 8b).

## 4 DISCUSSION

### 4.1 Constitutive mountain birch BVOC emissions and photosynthetic rate depending on age and variation between leaves

Leaves in this study measured without prior exposure to O_3_ represent the constitutive BVOC emissions from mountain birch (*Betula pubescens* ssp. *czerepanovii*). We found emission rates to depend on leaf age; early leaves emitted more SQTs and OXYs compared to late leaves, which instead emitted more MT. The age‐dependent differences in this study were not statistically significant, but they were in agreement with the study by Mofikoya et al. (2018), which found similar, significant differences. Early leaves in our study tended to have higher variability in emission profiles and were mainly dominated by linalool, limonene, α‐pinene, and β‐farnesene, compared to late leaves that mainly emitted α‐pinene (Table S1). The differences in emission profiles for leaf age were also found in Hellén et al. (2021), analyzing BVOC emissions from downy birch (*Betula pubescens*), finding early leaf emissions to be linalool‐ and SQT‐dominated and late leaves dominated by MTs. Measurements of constitutive mountain birch emission rates have been reported previously, and Haapanala et al. (2009) and Ahlberg (2011) measured leaf emissions in the same area with similar timing and temperature (20°C) as our late leaves. Our measured MT emissions are within the range from both studies (12 to 4000 ng g_dw_^−1^ h^−1^), while the SQT emissions in our study were only comparable to Ahlberg (2011) of 5.5 ng g_dw_^−1^ h^−1^, as higher SQT emissions ranging between 25 and 2700 ng g_dw_^−1^ h^−1^ were found by Haapanala et al. (2009). The lower SQT emissions in this study could potentially be explained by the experimental setup. Since we installed MnO_2_ nets before the sampling tubes to remove O_3_, there is a possibility that the SQT emissions are underestimated as these nets were found to scrub SQTs from the air (Hellén et al., 2023). Expectedly, we also found early leaves to have lower and more variable photosynthetic rates compared to late leaves, which were both higher and more stable with respect to photosynthesis (Bielczynski et al., 2017). The photosynthetic rates for the leaves were also in line with the previous study by Ahlberg (2011).

In addition to our findings that photosynthetic and BVOC emission rates change with leaf age, we also found large variations in both photosynthetic and BVOC emission rates within both age groups. Variations between trees are expected regardless of age, depending on the different chemotypes (Bäck et al., 2012; Haapanala et al., 2009). Not commonly reported are variations from leaves on the same branch of a tree, which was the case for the leaves in this study. One part of the variation between the early leaves could potentially be explained by the high emissions of α‐pinene from two leaves on Birch 3 (Figure S2a). A‐pinene was the dominant compound and emitted at higher rates from late leaves compared to early leaves, however, not significantly (Table S2). Based on these results, we hypothesize that the high emissions of α‐pinene from the two early leaves are an indication that they were more mature compared to the other early leaves. To confirm this, we repeated the statistical analysis comparing leaf age emissions while excluding early leaves from Birch 3. This resulted in significantly higher α‐pinene emissions in late summer compared to early leaves (*p* = .028). We do, however, not expect the varying maturity to contribute to the variations we saw for the late leaves; thus, another explanation could be sun exposure. BVOC emissions have been found to be affected by sunlit or shaded conditions (Karlsson et al., 2021; Keenan et al., 2011), and that could potentially explain the variations. During leaf selection, we did, however, take this into consideration and only chose leaves that were not directly sunlit upon measurements. Leaves from the same branch were also always at the same height and from the outer part, suggesting that sun exposure variations between leaves are low. Another limitation of the study design is the sampling method. Adsorbent tubes give the accumulated concentration during the measurement time, in our case for 1 h during O_3_ exposure and 30 min when not exposed. This means that quick adjustments by the leaves cannot be seen with this method. Other methods for quantifying rapid changes in BVOC emissions exist, such as a PTR‐ToF‐MS, and could facilitate fast adjustment measurements; however, using such instrumentation is limited in field studies similar to this one, and PTR‐techniques are not able to separate compounds that share the same mass, i.e. MT compounds cannot be distinguished. To determine the cause of the within‐branch leaf variability, more data and potentially different sampling methods would be needed. Our findings still highlight the importance of considering leaf‐scale measurements in addition to branch‐scale.

### 4.2 Exposure to O_3_ alters BVOC emissions depending on concentration level and leaf age

Trees have various ways of protection against oxidative stress from O_3_, for example, reducing stomatal conductance to avoid O_3_ from entering the leaf, exuding metabolites from glandular trichomes, or inducing BVOCs to react with O_3_ outside the leaf (Calfapietra et al., 2013; Li et al., 2018; Pääkkönen et al., 1996). When measuring the impact of O_3_ in this study, we focused on the emission rates of BVOCs and the direct response of these. Other protection mechanisms were not studied, but as glandular trichome density for mountain birch does not change when leaves are fully unfolded to mature (Valkama et al., 2004), this should not influence the results in O_3_ responses with leaf age.

When exposed to 40 ppb, early leaves did not change the total emission rate compared to the control, but we saw increases in emissions of the green leaf volatile (GLV) cis‐3‐hexenol (Figure 6a). Cis‐3‐hexenol is emitted through the LOX pathway in the leaf, and products from this pathway have been proven to be induced by O_3_ leaf damage (Li et al., 2018). We did not witness the same induction of cis‐3‐hexenol for late leaves at 40 ppb, but the emission rates were instead significantly lower at 40 ppb compared to the control, with an emission rate almost 20 times higher (Figure 6b). The significant response of the late leaves at 40 ppb was not expected since it is not uncommon that trees in Abisko experience similar ambient concentrations. Between 2004 and 2008, the maximum monthly O_3_ concentrations were between 32 ppb and 49 ppb on average in Latnjajaure, close to Abisko (Klingberg et al., 2009). Because of this, our expectation was that emission rates for late leaves at 40 ppb would be similar to the control. The reason the results differ from our expectation can be due to the method where control leaves were exposed to O_3_‐free air, common for chamber measurements of BVOCs (Ortega & Helmig, 2008). However, there is always some level of O_3_ in the ambient air, and the constitutive control emissions we found might instead be an overestimation of the emission rates from the birch leaves outside the enclosures, at least for late leaves.

The largest response to acute O_3_ exposure was from the early leaves on Birch 1 at 120 ppb O_3_ (Figure 5). The other birches acted differently for early leaves; Birch 3 responded similar to the late leaves, with decreased emission rates at O_3_ exposure and higher when not exposed. Birch 2 responded similarly to Birch 3, but had a different compound blend mainly dominated by SQT. We believed this to be due to differences in chemotype as previously found for mountain birch in Abisko (Haapanala et al., 2009); however, the same birch was not dominated by SQT later in the summer, indicating that this is not the case. This further highlights the variability of emission rates by age. Late leaves responded similarly to O_3_ exposure, with emission rates at comparable levels at all exposures, and did induce emissions as much as early leaves (Table S4). The compound group increasing the most for both ages was SQT at 120 ppb (Figures 5 and 6). This is similar to Bourtsoukidis et al. (2012), who found O_3_ stress to be a driver for SQT emissions from Norway spruce (*Picea abies*). In this study, the SQT emissions were mainly induced after exposure to 120 ppb but not after 80 ppb. Our findings thus suggest that a potential stress threshold lies between 80 and 120 ppb upon acute O_3_ exposure. Apart from SQT emission, cis‐3‐hexenol was already induced at 40 ppb for two of the birches during the early season; this indicates that early leaves might be more sensitive to O_3_ at lower concentrations that might not be seen as SQT induction (Figure 5a). One early birch did not respond with induced cis‐3‐hexenol at any step; it instead drastically induced α‐pinene emission rates after exposure to 120 ppb (Birch 3, Figure 4), something that could indicate accelerated senescence (Karlsson et al., 2003; Riikonen et al., 2004).

Based on the results, looking at the ratio of SQT:MT might indicate O_3_ stress, as an induction of SQT emission was seen with O_3_ stress (Figures 5 and 6). From the ratio, the O_3_ stress on early leaves through induced SQT is only seen at higher O_3_ concentrations (120 ppb), while late leaves respond with increased SQT:MT at each exposure (Figure 8). This indicates a faster defense response from late leaves. The late leaves could respond faster due to low‐concentration priming effects from ambient O_3_ concentrations (Li et al., 2017), as they have naturally been exposed to O_3_ for longer. This was also seen through photosynthesis, with steadier rates for late leaves throughout the experiment compared to early leaves. The early leaf photosynthesis rates also did not recover between the exposure stages (Table S3 and S4). The same was seen for the stomatal conductance, which indicated stress earlier for the early than the late leaves. This further suggests early leaves are more vulnerable to O_3_ stress than late leaves.

### 4.3 O_3_ ‐induced BVOC emission implications for the sub‐Arctic areas

The volatility and oxidative products of BVOCs determine the efficiency of growing SOA particles. Emitted compounds have different SOA yields; compound blend changes can then impact the locally produced SOA (Lee et al., 2006; Scott et al., 2018; Zhao et al., 2017). SOA formation capacity was enhanced by moth attacks on mountain birch (Rieksta et al., 2020; Yli‐Pirilä et al., 2016) by up to a factor of 5 lasting several years (Taipale et al., 2021; Ylivinkka et al., 2020). The main increases from moth outbreaks came from SQT and GLV, similar to inductions from elevated O_3_ in this study. Based on this, there is a potential that increased O_3_ stress could lead to increased SOA formation in addition to plant damage. We found early leaf α‐pinene emission rates to increase on average 15 times after exposure at 120 ppb compared to the control. A‐pinene effectively fosters particle growth, indicating further SOA yield potential with O_3_ exposure (Lee et al., 2006). There are further implications of high O_3_ concentrations in ambient air; BVOC compounds react faster in the atmosphere at high O_3_ concentrations compared to low concentrations (Atkinson & Arey, 2003; Masui et al., 2023). This could impair plant–plant communications through BVOCs, which for the sub‐Arctic area prone to moth outbreaks might have negative impacts as stress‐induced signals would not reach far from the emitting plant.

To conclude, future acute increases in tropospheric O_3_ over the sub‐Arctic can have large implications for mountain birch and their environment. Elevated O_3_ was more stressful for early leaves, indicating a potential for lower stress thresholds in early summer, a season already experiencing higher levels of O_3_. If those levels increase more, it would cause plant stress, affecting the photosynthetic rate of the mountain birch and inhibiting plant growth. There were indications of leaf senescence in the early leaves after exposure to 120 ppb, further highlighting the negative impact of elevated O_3_. Late leaves were not as sensitive to the elevated O3 concentration in this study and could have better protection against O_3_ stress, potentially due to a priming effect. However, stress‐induced signals from SQT were seen from late leaves after exposure at 120 ppb. If elevated tropospheric O_3_ becomes the norm in the sub‐Arctic, it might have further implications for the local climate and is thus important to consider and take measures to prevent this from happening.

## FUNDING INFORMATION

This project was supported by the Swedish Society for Anthropology and Geography and the Physiographic Society of Lund and by the BioDiv‐Support project funded through the 2017–2018 Belmont Forum and BiodivERsA joint call for research proposals, under the BiodivScen ERA‐Net COFUND program, and with the funding organizations AKA (contract no. 326328), ANR (ANR‐18‐EBI4‐0007), BMBF (KFZ: 01LC1810A), FORMAS (contract nos. 2018–02434, 2018–02436, 2018–02437, 2018–02438), and MICINN (through APCIN: PCI2018‐093149).

## CONFLICT OF INTEREST STATEMENT

The authors declare that they have no conflict of interest.

## Supporting information


Data S1.

## Data Availability

The data that support the findings of this study can be found in Zenodo at https://doi.org/10.5281/zenodo.10041138.
