# Supplementary material for: Ozone stress response of leaf BVOC emission and photosynthesis in mountain birch (Betula pubescens spp. czerepanovii) depends on leaf age
Source: Plant Environ Interact. 2024 Feb 4;5(1):e10134. doi: 10.1002/pei3.10134 (PMC10840370; doi:10.1002/pei3.10134)
Supplement: Supplementary file 1 — Data S1. [file PEI3-5-e10134-s001.docx]

## Supporting Information

Article title: Ozone stress response of leaf BVOC emission and photosynthesis in mountain birch (*Betula pubescens* spp. *czerepanovii*) depends on leaf age

Authors: Erica Jaakkola et al.

The following Supporting Information is available for this article:

**Table S1.** The dominating compounds with the largest and second to largest contribution to the emission blends for each non-exposed birch leaf. The emission contribution (%) is calculated as the emission of the emitted compounds divided by the sum of emissions from all compounds. The leaves individual id is named BXLX, where B is short for birch/branch and L is short for leaf, X is the specific number. Leaves with the same BX number is always from the same branch on the same birch. The compound MBO is abbreviated from 2-methyl-3-buten-1-ol.

**Table S2.** The mean BVOC emission rate and standard deviation and their limit of detection (LOD; ng g_dw_^-1^ h^-1^) for all individual compounds for the healthy leaves during both early (n = 10) and late (n = 11) summer. Presented is also the total emission rate as summed mean of the compound, the mean photosynthetic rate (µmol m^-2^ s^-1^), mean transpiration rate (mmol m^-2^ s^-1^), mean stomatal conductance (mol H_2_O m^-2^ s^-1^), mean specific leaf area (SLA; cm^2^ g^-1^) and mean chlorophyll content (µg cm^-2^). The table also reveals the results of the Kruskal-Wallis test when comparing the early and late season for each variable. Statistically significant differences (*P* < 0.05) are marked with **. The abbreviated compounds are 4-acetyl-1-methylcyclohexane (AMCH) and 2-methyl-3-buten-1-ol (MBO).

**Table S3.** The average total BVOC emission (ng g_dw_^-1^ h^-1^) and photosynthetic rate (µmol m^-2^ s^-1^) and respective standard deviation for each birch branch in their healthy state and during the recovery phases of the experiment sequence for both the early and late summer measurements. The emission rate is the sum of the mean of the compounds emitted from each leaf over the birch branches. The leaves are exposed to 0 ppb of ozone during the healthy state and the recovery phases and recovery phase 1 is after an exposure of 40 ppb, recovery phase 2 after 80 ppb and recovery phase 3 after 120 ppb.

**Table S4.** The average total BVOC emission (ng g_dw_^-1^ h^-1^) and photosynthetic rate (µmol m^-2^ s^-1^) and respective standard deviation for each birch branch in their healthy state and during the ozone exposure phases of the experiment sequence for both the early and late summer measurements. The emission rate is the sum of the mean of the compounds emitted from each leaf over the birch branches. The leaves are exposed to 0 ppb of ozone during the healthy state and later exposed to 40 ppb, 80 ppb and 120 ppb with recovery phases in between.

**Figure S1.** Stomatal conductance for the leaves measured in early and late summer for each step in the measurement sequence of exposure to 0 ppb O_3_ or the respective elevated concentrations given by the plot. The mean ± standard deviation is visualized by the circle and whiskers.

**Figure S2.** The emission rate for the quantified BVOC compounds and the respective photosynthetic rate for each measured non-exposed mountain birch leaf. Presented is (a) the early summer emission rates and photosynthetic rate, (b) the early summer the BVOC emission blend, (c) the late summer emission rate and photosynthetic rate and (d) the late summer BVOC emission blend. Note that the left-side y-axis is presented in a logarithmic scale for comparability. The leaves individual id is named BXLX, where B is short for birch/branch and L is short for leaf, X is the specific number. Leaves with the same BX number is always from the same branch on the same birch. The BVOC compounds are separated by color in their respective groups with monoterpenes (MT) in red-pink, sesquiterpene (SQT) in greens and oxygenated compounds (OXY) in blues, where MBO is abbreviated from 2-methyl-3-buten-1-ol and AMCH from 4-acetyl-1-methylcyclohexene. Isoprene is separated on its own and colored in yellow.

**Figure S3.** The recovery-phase birch branches’ (a) early summer BVOC emission blend during recovery phase 1, (b) early summer BVOC emission blend during recovery phase 2, (c) early summer BVOC emission blend during recovery phase 3, (d) late summer BVOC emission blend during recovery phase 1, (e) late summer BVOC emission blend during recovery phase 2 and (f) late summer BVOC emission blend during recovery phase 3. The birch leaves were exposed to elevated ozone at concentrations of 40 ppb, 80 ppb and 120 ppb prior to measuring the respective recovery phase 1-3 at an exposure concentration of 0 ppb. The BVOC compounds are separated by color in their respective groups with monoterpenes (MT) in red-pink, sesquiterpene (SQT) in greens and oxygenated compounds (OXY) in blues, where MBO is abbreviated from 2-methyl-3-buten-1-ol and AMCH from 4-acetyl-1-methylcyclohexene. Isoprene is separated on its own and colored in yellow. **Figure S3.** The ozone exposure-phase birch branches’ (a) early summer BVOC emission blend at 40 ppb, (b) early summer BVOC emission blend at 80 ppb, (c) early summer BVOC emission blend at 120 ppb, (d) late summer BVOC emission blend at 40 ppb, (e) late summer BVOC emission blend at 80 ppb and (f) late summer BVOC emission blend at 120 ppb. The birch leaves were exposed to elevated ozone at concentrations of 40 ppb, 80 ppb and 120 ppb following a recovery phase of 0 ppb. The BVOC compounds are separated by color in their respective groups with monoterpenes (MT) in red-pink, sesquiterpene (SQT) in greens and oxygenated compounds (OXY) in blues, where MBO is abbreviated from 2-methyl-3-buten-1-ol and AMCH from 4-acetyl-1-methylcyclohexene. Isoprene is separated on its own and colored in yellow.

**Figure S4.** The ozone exposure-phase birch branches’ (a) early summer BVOC emission blend at 40 ppb, (b) early summer BVOC emission blend at 80 ppb, (c) early summer BVOC emission blend at 120 ppb, (d) late summer BVOC emission blend at 40 ppb, (e) late summer BVOC emission blend at 80 ppb and (f) late summer BVOC emission blend at 120 ppb. The birch leaves were exposed to elevated ozone at concentrations of 40 ppb, 80 ppb and 120 ppb following a recovery phase of 0 ppb. The BVOC compounds are separated by color in their respective groups with monoterpenes (MT) in red-pink, sesquiterpene (SQT) in greens and oxygenated compounds (OXY) in blues, where MBO is abbreviated from 2-methyl-3-buten-1-ol and AMCH from 4-acetyl-1-methylcyclohexene. Isoprene is separated on its own and colored in yellow..

**Figure S5.** Statistically significant differences between the steps in the measurement sequence for the early summer photosynthetic rate, total emission rate and all the identified compounds. A dark blue box indicates a significant difference between the healthy/exposure/recovery phases. Statistical analysis was performed using a Kruskal-Wallis test followed by a multiple comparisons procedure with Dunn-Sidák’s approach to see which groups were different from each other. The compound MBO is abbreviated from 2-methyl-3-buten-1-ol and AMCH from 4-acetyl-1-methylcyclohexene

**Figure S6.** Statistically significant differences between the steps in the measurement sequence for the late summer photosynthetic rate, total emission rate and all the identified compounds. A dark blue box indicates a significant difference between the healthy/exposure/recovery phases. Statistical analysis was performed using a Kruskal-Wallis test followed by a multiple comparisons procedure with Dunn-Sidák’s approach to see which groups were different from each other. The compound MBO is abbreviated from 2-methyl-3-buten-1-ol and AMCH from 4-acetyl-1-methylcyclohexene

**Table S1.** The dominating compounds with the largest and second to largest contribution to the emission blends for each non-exposed birch leaf. The emission contribution (%) is calculated as the emission of the emitted compounds divided by the sum of emissions from all compounds. The leaves individual id is named BXLX, where B is short for birch/branch and L is short for leaf, X is the specific number. Leaves with the same BX number is always from the same branch on the same birch. The compound MBO is abbreviated from 2-methyl-3-buten-1-ol.

| Leaf ID | Time | 1st compound | Emission contribution (%) | 2nd compound | Emission contribution (%) |
| --- | --- | --- | --- | --- | --- |
| B1L1 | Early | Linalool | 70 | cis-3-Hexenol | 20 |
| B1L2 | Early | Limonene | 33 | β-Caryophyllene | 20 |
| B1L3 | Early | Isoprene | 91 | β-Pinene | 9 |
| B2L1 | Early | Linalool | 45 | Isoprene | 34 |
| B2L2 | Early | β-Farnesene | 92 | Nopinone | 8 |
| B2L3 | Early | Linalool | 92 | Nopinone | 8 |
| B2L5 | Early | Linalool | 67 | cis-3-Hexenol | 18 |
| B3L1 | Early | Limonene | 20 | Linalool | 19 |
| B3L2 | Early | α-Pinene | 85 | Camphene | 6 |
| B3L3 | Early | α-Pinene | 43 | p-Cymene | 16 |
| B1L3 | Late | α-Pinene | 55 | Linalool | 36 |
| B1L4 | Late | α-Pinene | 93 | Carene | 4 |
| B1L5 | Late | α-Pinene | 66 | Limonene | 14 |
| B1L6 | Late | cis-3-Hexenol | 60 | p-Cymene | 30 |
| B2L1 | Late | α-Pinene | 29 | MBO | 23 |
| B2L2 | Late | α-Pinene | 86 | β-Farnesene | 6 |
| B2L3 | Late | α-Pinene | 76 | Limonene | 9 |
| B3L1 | Late | Camphene | 42 | Carene | 37 |
| B3L2 | Late | α-Pinene | 32 | Camphene | 21 |
| B3L3 | Late | α-Pinene | 42 | Limonene | 17 |
| B3L4 | Late | α-Pinene | 68 | Camphene | 14 |

**Table S2.** The mean BVOC emission rate and standard deviation and their limit of detection (LOD; ng g_dw_^-1^ h^-1^) for all individual compounds for the healthy leaves during both early (n = 10) and late (n = 11) summer. Presented is also the total emission rate as summed mean of the compound, the mean photosynthetic rate (µmol m^-2^ s^-1^), mean transpiration rate (mmol m^-2^ s^-1^), mean stomatal conductance (mol H_2_O m^-2^ s^-1^), mean specific leaf area (SLA; cm^2^ g^-1^) and mean chlorophyll content (µg cm^-2^). The table also reveals the results of the Kruskal-Wallis test when comparing the early and late season for each variable. Statistically significant differences (*P* < 0.05) are marked with **. The abbreviated compounds are 4-acetyl-1-methylcyclohexane (AMCH) and 2-methyl-3-buten-1-ol (MBO).

|  | Early summer (mean) | Early summer (SD) | Late summer (mean) | Late summer (SD) | P |  | LOD | Unit |
| --- | --- | --- | --- | --- | --- | --- | --- | --- |
| BVOC compounds | | | | | | |  |  |
| AMCH | 0 | 0 | 1.4 | 2.3 | 0.48 |  | 0.89 | ng g_dw_^-1^ h^-1^ |
| Isoprene | 13.6 | 17 | 6.4 | 8 | 0.56 |  | 10.3 | ng g_dw_^-1^ h^-1^ |
| MBO | 2.2 | 3.6 | 15.9 | 25 | 0.22 |  | 6.73 | ng g_dw_^-1^ h^-1^ |
| α-Humulene | 0.3 | 0.6 | 0.9 | 0.7 | 0.15 |  | 0.89 | ng g_dw_^-1^ h^-1^ |
| α-Pinene | 52.5 | 104.4 | 357.3 | 785.2 | 0.05 |  | 0.89 | ng g_dw_^-1^ h^-1^ |
| β-Caryophyllene | 15.7 | 28 | 1.6 | 1.6 | 0.69 |  | 0.45 | ng g_dw_^-1^ h^-1^ |
| β-Farnesene | 8.2 | 9.5 | 1.3 | 3.3 | 0.07 |  | 5.3 | ng g_dw_^-1^ h^-1^ |
| β-Pinene | 1.2 | 1.3 | 3.9 | 8.8 | 0.6 |  | 1.8 | ng g_dw_^-1^ h^-1^ |
| Camphene | 4.6 | 8.2 | 28.1 | 28.8 | 0.02 | ** | 0.45 | ng g_dw_^-1^ h^-1^ |
| Carene | 7.7 | 10.7 | 16.8 | 30.6 | 0.52 |  | 0.45 | ng g_dw_^-1^ h^-1^ |
| Cis-3-Hexenol | 16.5 | 25.9 | 4.2 | 4.5 | 0.51 |  | 1.35 | ng g_dw_^-1^ h^-1^ |
| Isolongifolene | 1.6 | 2.1 | 0 | 0 | 0.49 |  | 1.35 | ng g_dw_^-1^ h^-1^ |
| Limonene | 27.9 | 40.3 | 18.3 | 26.9 | 0.66 |  | 3.6 | ng g_dw_^-1^ h^-1^ |
| Linalool | 54.5 | 75.9 | 2.9 | 4.3 | 0.07 |  | 1.34 | ng g_dw_^-1^ h^-1^ |
| Longicyclene | 3 | 3.6 | 0.1 | 0.3 | 0.002 | ** | 0.45 | ng g_dw_^-1^ h^-1^ |
| Nopinone | 5.9 | 7.4 | 1.5 | 0.4 | 0.48 |  | 0.89 | ng g_dw_^-1^ h^-1^ |
| p-Cymene | 11.6 | 27 | 11.7 | 15.7 | 0.27 |  | 0.45 | ng g_dw_^-1^ h^-1^ |
| Total emission rate | 189 | 189 | 456 | 876 | 0.57 |  |  | ng g_dw_^-1^ h^-1^ |
| Physiological parameters | | | | | | |  |  |
| Photosynthetic rate | 11.1 | 4.6 | 13.9 | 2 | 0.02 | ** |  | µmol m^-2^ s^-1^ |
| Transpiration rate | 1.3 | 0.55 | 1.8 | 0.59 | < 0.001 | ** |  | mmol m^-2^ s^-1^ |
| Stomatal conductance | 0.11 | 0.05 | 0.19 | 0.06 | < 0.001 | ** |  | mol H_2_O m^-2^ s^-1^ |
| Leaf traits | | | | | | |  |  |
| SLA | 144 | 20 | 132 | 12 | < 0.001 | ** |  | cm^2^ g^-1^ |
| Chlorophyll content | 34 | 2 | 39 | 2 | < 0.001 | ** |  | µg cm^-2^ |

**Table S3.** The average total BVOC emission (ng g_dw_^-1^ h^-1^) and photosynthetic rate (µmol m^-2^ s^-1^) and respective standard deviation for each birch branch in their healthy state and during the recovery phases of the experiment sequence for both the early and late summer measurements. The emission rate is the sum of the mean of the compounds emitted from each leaf over the birch branches. The leaves are exposed to 0 ppb of ozone during the healthy state and the recovery phases and recovery phase 1 is after an exposure of 40 ppb, recovery phase 2 after 80 ppb and recovery phase 3 after 120 ppb.

|  | BVOC (ng g_dw_^-1^ h^-1^) | | | | Photosynthesis (µmol m^-2^ s^-1^) | | | |
| --- | --- | --- | --- | --- | --- | --- | --- | --- |
|  | Healthy | Recovery phase 1 | Recovery phase 2 | Recovery phase 3 | Healthy | Recovery phase 1 | Recovery phase 2 | Recovery phase 3 |
| Early | sum of mean (SD) | sum of mean (SD) | sum of mean (SD) | sum of mean (SD) | sum of mean (SD) | Mean (SD) | Mean (SD) | Mean (SD) |
| Birch 1 | 240  (26) | 131  (24) | 96  (16) | 103  (16) | 10  (3) | 12  (9) | 7  (7) | 11  (1) |
| Birch 2 | 70  (9) | 112  (16) | 94  (9) | 125  (12) | 11  (3) | 10  (2) | 8  (1) | 7  (0) |
| Birch 3 | 297  (39) | 81  (6) | 139  (12) | 2508  (528) | 12  (2) | 12  (1) | 10  (2) | 10  (2) |
| Average | 227  (17) | 128  (13) | 136  (11) | 939  (182) | 11  (5) | 11  (2) | 8  (4) | 9  (2) |
| Late |  |  |  |  |  |  |  |  |
| Birch 1 | 934  (196) | 769  (171) | 487  (98) | 261  (53) | 13  (2) | 14  (1) | 12  (2) | 12  (2) |
| Birch 2 | 236  (33) | 151  (30) | 176  (34) | 151  (32) | 10  (2) | 11  (1) | 10  (2) | 10  (2) |
| Birch 3 | 143  (14) | 139  (24) | 226  (40) | 232  (39) | 15  (1) | 16  (1) | 16  (2) | 15  (1) |
| Average | 470  (85) | 380  (81) | 330  (61) | 232  (42) | 14  (2) | 13  (2) | 13  (3) | 12  (3) |

**Table S4.** The average total BVOC emission (ng g_dw_^-1^ h^-1^) and photosynthetic rate (µmol m^-2^ s^-1^) and respective standard deviation for each birch branch in their healthy state and during the ozone exposure phases of the experiment sequence for both the early and late summer measurements. The emission rate is the sum of the mean of the compounds emitted from each leaf over the birch branches. The leaves are exposed to 0 ppb of ozone during the healthy state and later exposed to 40 ppb, 80 ppb and 120 ppb with recovery phases in between.

|  | BVOC (ng g_dw_^-1^ h^-1^) | | | | Photosynthesis (µmol m^-2^ s^-1^) | | | |
| --- | --- | --- | --- | --- | --- | --- | --- | --- |
|  | Healthy | 40 ppb | 80 ppb | 120 ppb | Healthy | 40 ppb | 80 ppb | 120 ppb |
| Early | sum of mean (SD) | sum of mean (SD) | sum of mean (SD) | sum of mean (SD) | sum of mean (SD) | Mean (SD) | Mean (SD) | Mean (SD) |
| Birch 1 | 240  (26) | 133  (15) | 29  (5) | 562  (71) | 10  (3) | 12  (2) | 9  (8) | 10  (1) |
| Birch 2 | 70  (9) | 206  (43) | 41  (7) | 52  (7) | 11  (3) | 8  (1) | 8  (1) | 7  (1) |
| Birch 3 | 297  (39) | 230  (28) | 36  (3) | 34  (3) | 12  (2) | 12  (1) | 12  (2) | 9  (2) |
| Average | 227  (17) | 292  (41) | 55  (4) | 356  (38) | 11  (5) | 10  (2) | 10  (4) | 9  (2) |
| Late |  |  |  |  |  |  |  |  |
| Birch 1 | 934  (196) | 21  (2) | 2  (0) | 11  (1) | 13  (2) | 14  (2) | 13  (2) | 12  (2) |
| Birch 2 | 236  (33) | 16  (1) | 22  (4) | 20  (3) | 10  (2) | 11  (3) | 11  (3) | 10  (2) |
| Birch 3 | 143  (14) | 22  (2) | 13  (2) | 25  (3) | 15  (1) | 16  (1) | 16  (2) | 15  (2) |
| Average | 470  (85) | 25  (2) | 16  (2) | 22  (2) | 14  (2) | 14  (3) | 13  (3) | 13  (3) |


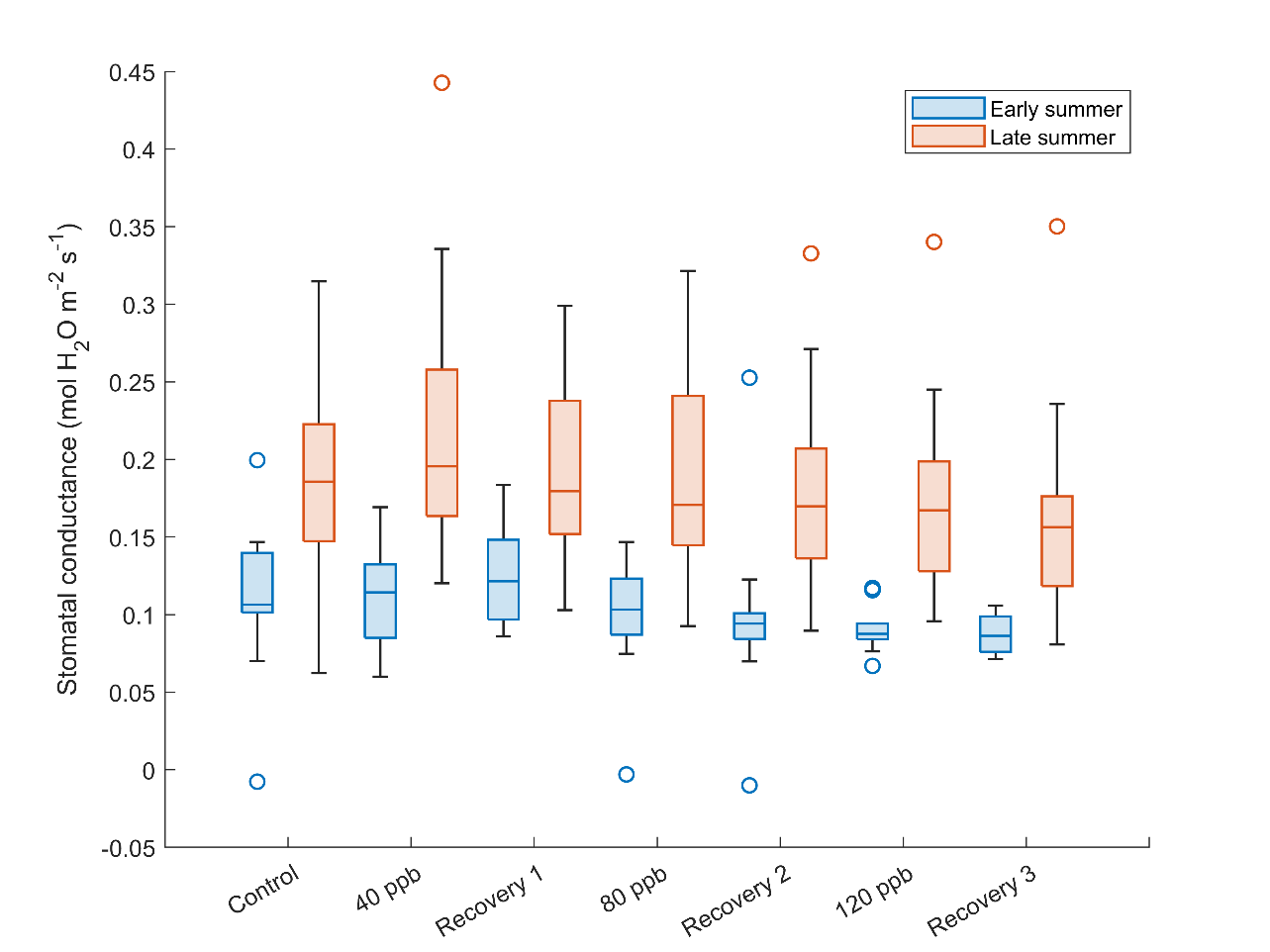


**Figure S1.** Stomatal conductance for the leaves measured in early and late summer for each step in the measurement sequence of exposure to 0 ppb O_3_ and the respective elevated concentrations of the measurement sequence.


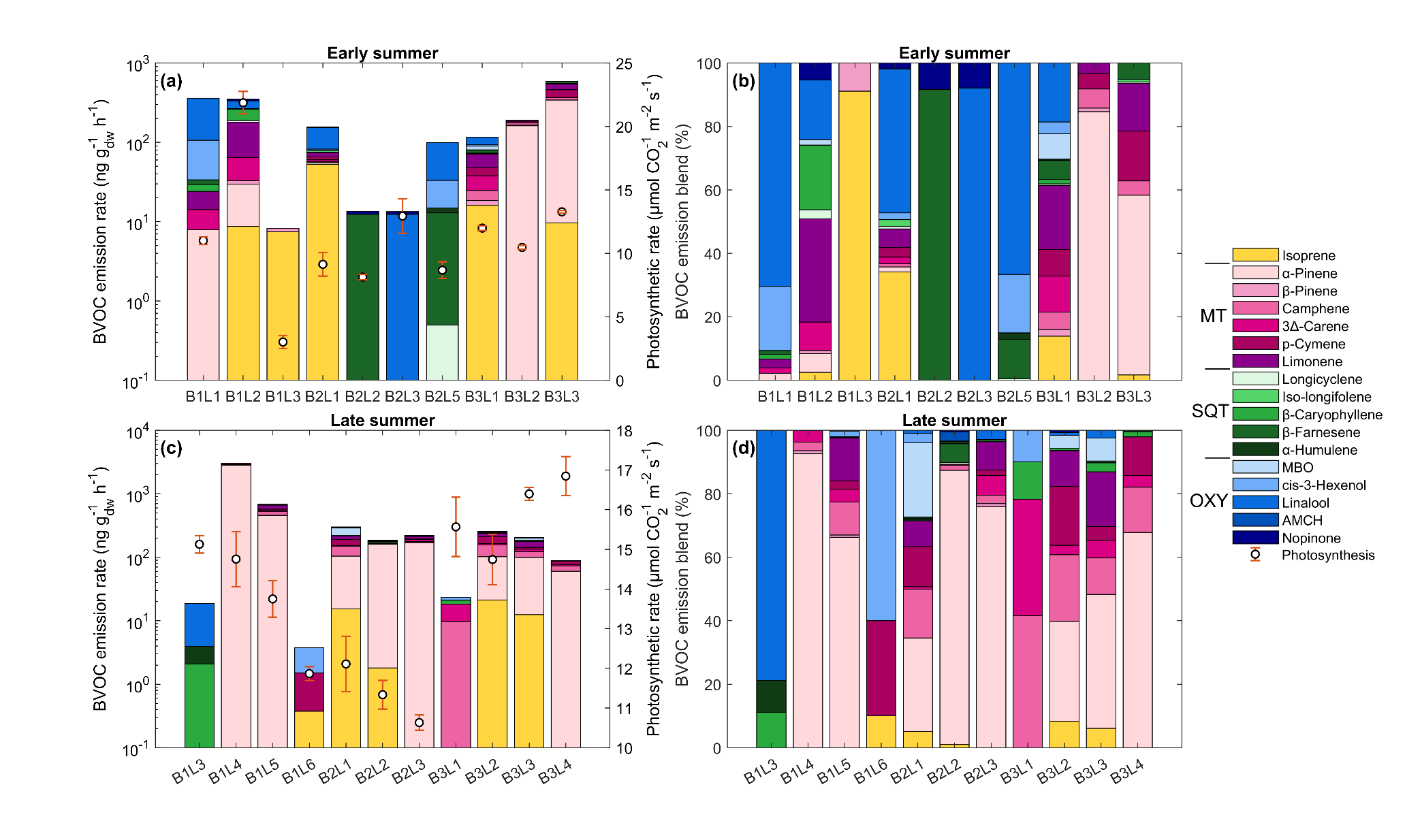


**Figure S2.** The emission rate for the quantified BVOC compounds, in log-scale for visual reasons, and the respective photosynthetic rate for each measured non-exposed mountain birch leaf. Presented is (a) the early summer emission rates and photosynthetic rate, (b) the early summer the BVOC emission blend, (c) the late summer emission rate and photosynthetic rate and (d) the late summer BVOC emission blend. Note that the left-side y-axis is presented in a logarithmic scale for comparability. The leaves individual id is named BXLX, where B is short for birch/branch and L is short for leaf, X is the specific number. Leaves with the same BX number is always from the same branch on the same birch. The BVOC compounds are separated by color in their respective groups with monoterpenes (MT) in red-pink, sesquiterpene (SQT) in greens and oxygenated compounds (OXY) in blues, where MBO is abbreviated from 2-methyl-3-buten-1-ol and AMCH from 4-acetyl-1-methylcyclohexene. Isoprene is separated on its own and colored in yellow.


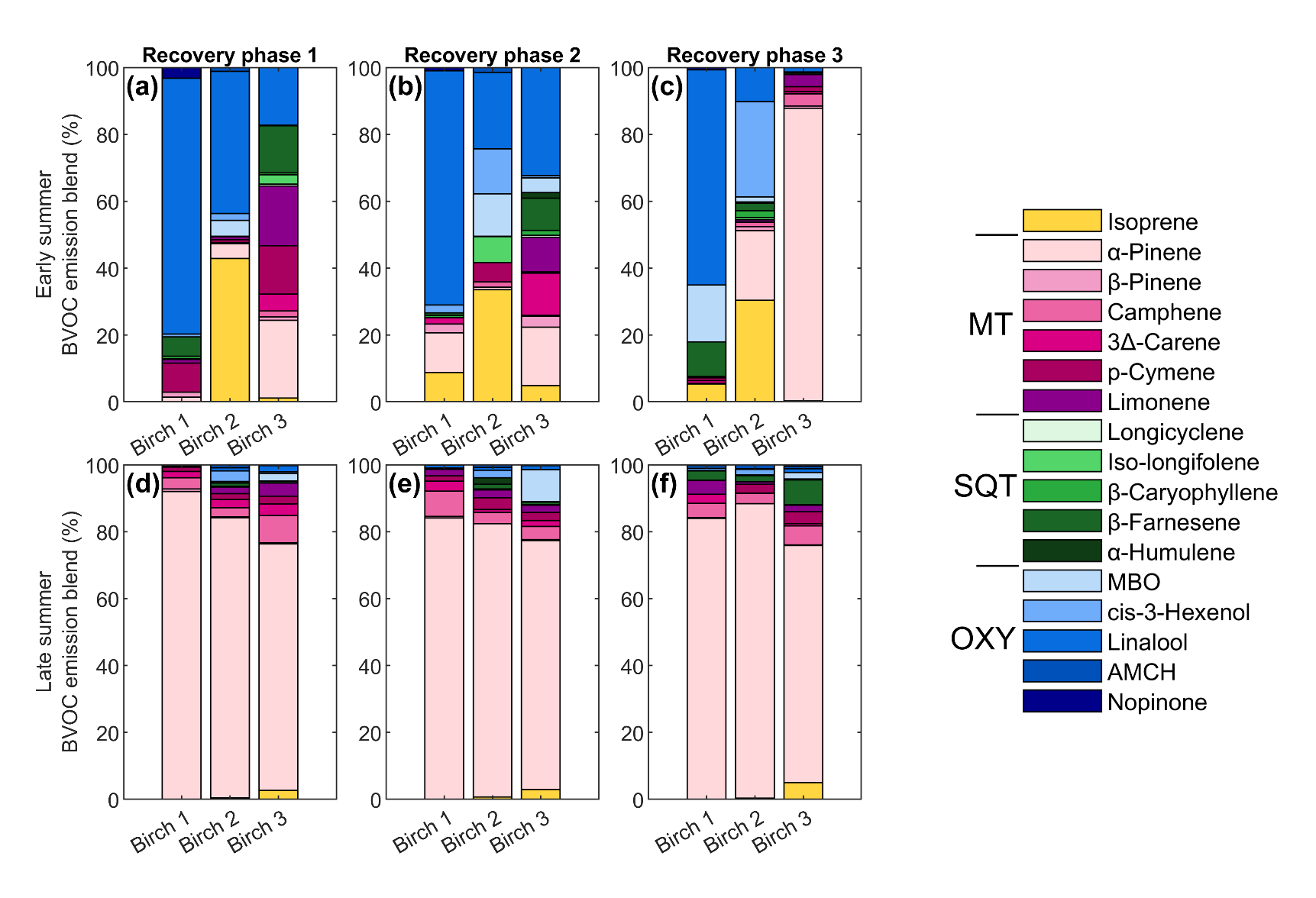


**Figure S3.** The recovery-phase birch branches’ (a) early summer BVOC emission blend during recovery phase 1, (b) early summer BVOC emission blend during recovery phase 2, (c) early summer BVOC emission blend during recovery phase 3, (d) late summer BVOC emission blend during recovery phase 1, (e) late summer BVOC emission blend during recovery phase 2 and (f) late summer BVOC emission blend during recovery phase 3. The birch leaves were exposed to elevated ozone at concentrations of 40 ppb, 80 ppb and 120 ppb prior to measuring the respective recovery phase 1-3 at an exposure concentration of 0 ppb. The BVOC compounds are separated by color in their respective groups with monoterpenes (MT) in red-pink, sesquiterpene (SQT) in greens and oxygenated compounds (OXY) in blues, where MBO is abbreviated from 2-methyl-3-buten-1-ol and AMCH from 4-acetyl-1-methylcyclohexene. Isoprene is separated on its own and colored in yellow.


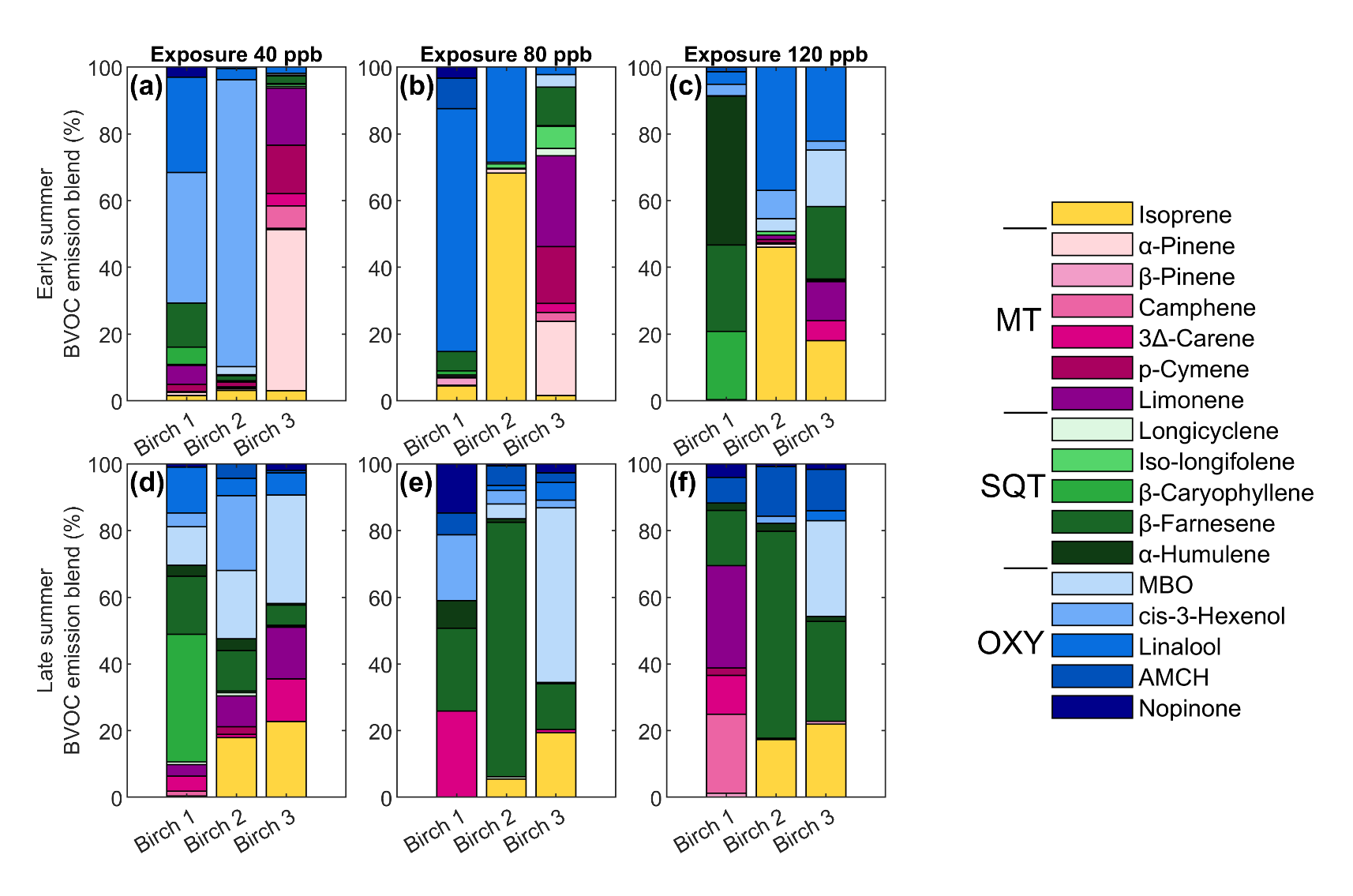


**Figure S4.** The ozone exposure-phase birch branches’ (a) early summer BVOC emission blend at 40 ppb, (b) early summer BVOC emission blend at 80 ppb, (c) early summer BVOC emission blend at 120 ppb, (d) late summer BVOC emission blend at 40 ppb, (e) late summer BVOC emission blend at 80 ppb and (f) late summer BVOC emission blend at 120 ppb. The birch leaves were exposed to elevated ozone at concentrations of 40 ppb, 80 ppb and 120 ppb following a recovery phase of 0 ppb. The BVOC compounds are separated by color in their respective groups with monoterpenes (MT) in red-pink, sesquiterpene (SQT) in greens and oxygenated compounds (OXY) in blues, where MBO is abbreviated from 2-methyl-3-buten-1-ol and AMCH from 4-acetyl-1-methylcyclohexene. Isoprene is separated on its own and colored in yellow..

**
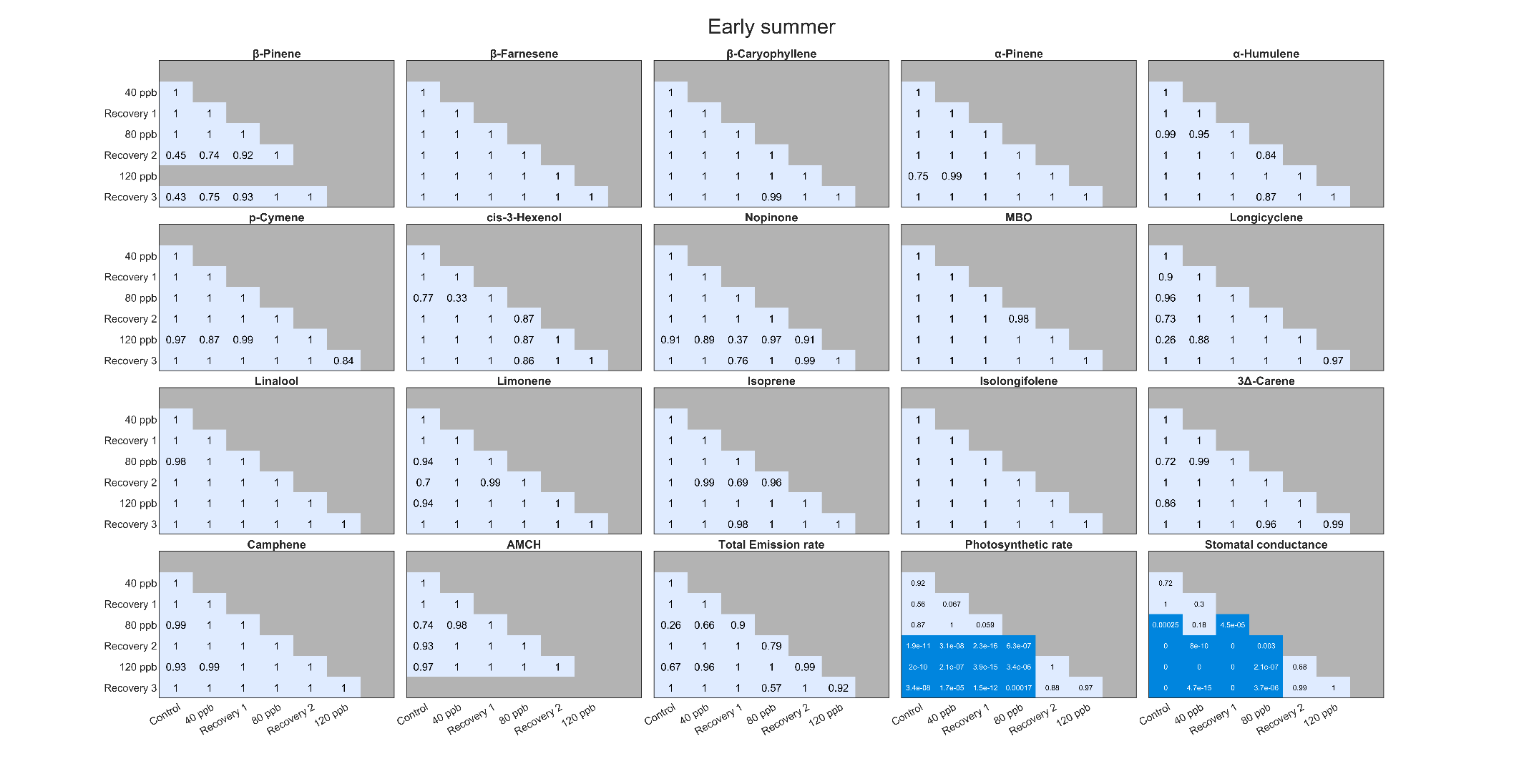
**

**Figure S5.** Statistically significant differences between the steps in the measurement sequence for the early summer photosynthetic rate, total emission rate and all the identified compounds. A dark blue box indicates a significant difference between the healthy/exposure/recovery phases. Statistical analysis was performed using a Kruskal-Wallis test followed by a multiple comparisons procedure with Dunn-Sidák’s approach to see which groups were different from each other. The compound MBO is abbreviated from 2-methyl-3-buten-1-ol and AMCH from 4-acetyl-1-methylcyclohexene

**
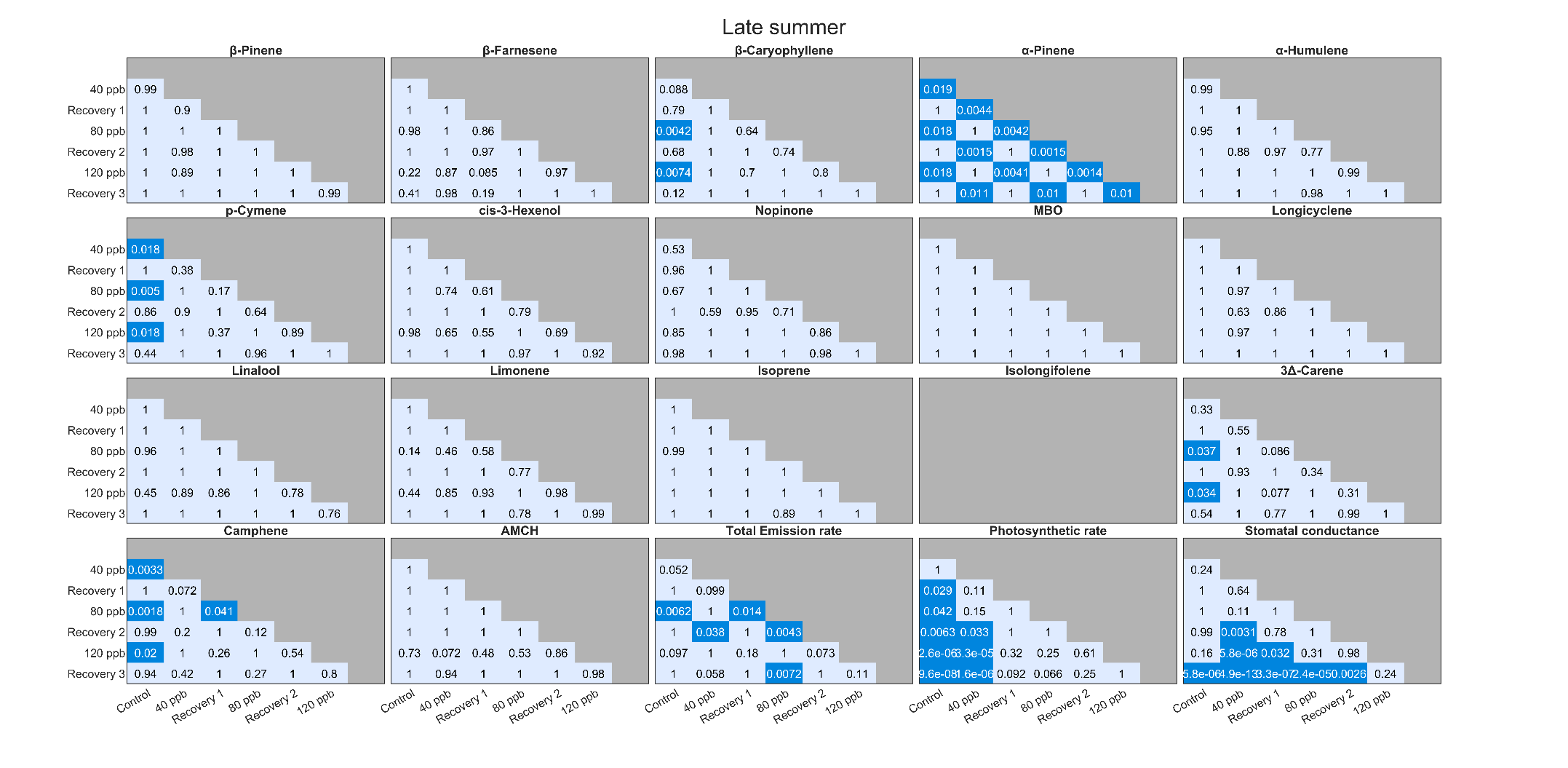
**

**Figure S6.** Statistically significant differences between the steps in the measurement sequence for the late summer photosynthetic rate, total emission rate and all the identified compounds. A dark blue box indicates a significant difference between the healthy/exposure/recovery phases. Statistical analysis was performed using a Kruskal-Wallis test followed by a multiple comparisons procedure with Dunn-Sidák’s approach to see which groups were different from each other. The compound MBO is abbreviated from 2-methyl-3-buten-1-ol and AMCH from 4-acetyl-1-methylcyclohexene
